# Recent Progresses in the Multicomponent Synthesis of Dihydropyridines by Applying Sustainable Catalysts Under Green Conditions

**DOI:** 10.3389/fchem.2021.800236

**Published:** 2021-12-22

**Authors:** Immandhi Sai Sonali Anantha, Nagaraju Kerru, Suresh Maddila, Sreekantha B. Jonnalagadda

**Affiliations:** ^1^ Department of Chemistry, GITAM Institute of Sciences, GITAM University, Visakhapatnam, India; ^2^ Department of Chemistry, GITAM School of Science, GITAM University, Bengaluru, India; ^3^ School of Chemistry and Physics, University of KwaZulu-Natal, Westville Campus, Chiltern Hills, Durban, South Africa

**Keywords:** green synthesis, 1,4-dihydropyridines, heterogeneous catalyst, multi-component reactions, one-pot synthesis

## Abstract

The synthesis of dihydropyridines, valuable molecules with diverse therapeutic properties, using eco-friendly heterogeneous catalysts as a green alternative received significant consideration. By selecting appropriate precursors, these compounds can be readily modified to induce the desired properties in the target product. This review focused on synthesising diverse dihydropyridine derivatives in single-pot reactions using magnetic, silica, and zirconium-based heterogeneous catalytic systems. The monograph describes preparation techniques for various catalyst materials in detail. It covers facile and benign magnetic, silica, zirconium-based, and ionic liquid catalysts, exhibiting significant efficacy and consistently facilitating excellent yields in short reaction times and in a cost-effective way. Most of the designated protocols employ Hantzsch reactions involving substituted aldehydes, active methylene compounds, and ammonium acetate. These reactions presumably follow Knoevenagel condensation followed by Michael addition and intra-molecular cyclisation. The multicomponent one-pot protocols using green catalysts and solvents have admirably increased the product selectivity and yields while minimising the reaction time. These sustainable catalyst materials retain their viability for several cycles reducing the expenditure are eco-friendly.

## Introduction

Synthesis of small heterocyclic organic molecules has advanced a long way with numerous applications in the academic and pharmaceutical industry, which are also valuable synthons for preparing elaborate bioactive molecules (Rotstein et al., 2014). The strategy of sustainable green methodologies for novel functionalised heterocyclic scaffolds is receiving substantial attention (Védrine, 2017; Kerru et al., 2019a; Bhaskaruni et al., 2020; Kerru et al., 2021a). Over the past two decades, heterogeneous catalysts have gained significant importance in synthetic organic chemistry due to their tunable acid–base properties, flexible textural characteristics, recyclability, excellent thermal stability, easy accessibility, and nontoxicity (Hutchings, 2009; José Climent et al., 2012; Maddila et al., 2020c; Devi et al., 2020; Ganta et al., 2021). Various materials have been explored as heterogeneous catalysts for synthesising small value-added heterocycles with improved atom efficiency and selectivity (Kerru et al., 2020a; Kerru et al., 2020b; Kerru et al., 2020c; Kerru et al., 2020i; Kerru et al., 2021b). These studies have provided insight for developing novel materials and understanding the reactivity and reaction mechanisms allowing the synthetic chemists to apply their innovations in scheming structures of organic synthesis reactions and utilise these molecules of potential activity (Rao et al., 2021a; Kerru et al., 2021c; Muralidhar et al., 2021; Suryanarayana et al., 2021).

The importance of sustainable heterogeneous catalysts and fiscal benefits is renascent through effective fusion procedures, which are deemed crucial in advanced organic chemistry (Kerru et al., 2020d; Kerru et al., 2020e; Kerru et al., 2020f). Recently, multicomponent reactions (MCRs) have been accepted as a significantly powerful tool in medicinal chemistry and thereby drug development programs (Slobbe et al., 2012; Maddila et al., 2020a). MCRs allow the building of multiple bonds and functionalised scaffolds in a one-pot manner, thus boosting efficiency and minimising the generation of by-products and purification techniques, meeting the green chemistry criteria (Maddila et al., 2020b; Maddila et al., 2020c; Devi et al., 2020). Using green principles has been gaining prominence in sustainable/combinatorial chemistry due to the global urge to enhance green credentials on production (Cioc et al., 2014; Shahid et al., 2017; Mokhtar et al., 2021). Sustainable methodologies by the MCR approach driven by green chemistry and green solvents, replacing the volatile and toxic solvents, are crucial in organic synthesis (Sheldon, 2005; Gu, 2012; Ferrazzano et al., 2019). Increasing environmental awareness drives designing of viable green protocols to gain prominence in organic synthesis (Kerru et al., 2019b; Maddila et al., 2019; Kerru et al., 2020g; Kerru et al., 2020h; Kerru et al., 2020j).

Nitrogen-based heterocyclic skeletons have prominence in synthetic organic chemistry because of their valuable biological applications (Kerru et al., 2019c; Kerru et al., 2020k; Kerru et al., 2020l; Rao et al., 2021b; Kumar et al., 2021). Among the several nitrogen heterocycles, dihydropyridines have received enormous attention due to their wide range of therapeutic properties. Such properties include calcium channel blocking, anti-mycobacterial, antioxidant, anti-dyslipidemic, Alzheimer’s disease, and antidiabetic activities (Kumar et al., 2010; Sirisha et al., 2011; Niaz et al., 2015; Schaller et al., 2018; Malek et al., 2019). Because of the growing reputation of dihydropyridines in the pharmaceutical field, numerous MCRs have been designed to synthesise 1,4-dihydropyridine scaffolds by utilising various heterogeneous catalysts (da Costa Cabrera et al., 2019; Rajesh et al., 2013; Mahinpour et al., 2018; Heydari et al., 2016; Bhaskaruni et al., 2017; Balaboina et al., 2019). Thus, the new MCRs with green protocols have drawn considerable interest, mainly in organic synthesis, drug development, and material science areas with green approaches using effective recyclable heterogeneous catalysts, sustainable procedures with remarkable atom, and carbon efficiency as added benefits. The review summarises the advances in constructing various dihydropyridine frameworks through the MCR approach by utilising diverse, robust heterogeneous catalysts.

## Synthesis of Dihydropyridines

**SCHEME 1:**
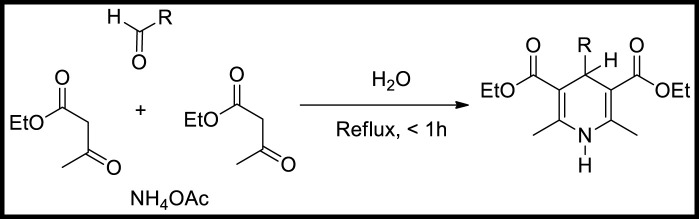
Hantzsch dihydropyridine synthesis.

**SCHEME 2:**
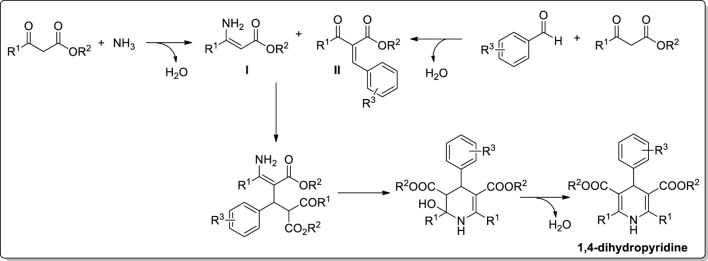
Synthetic route of dihydropyridine formation.

**FIGURE 1:**
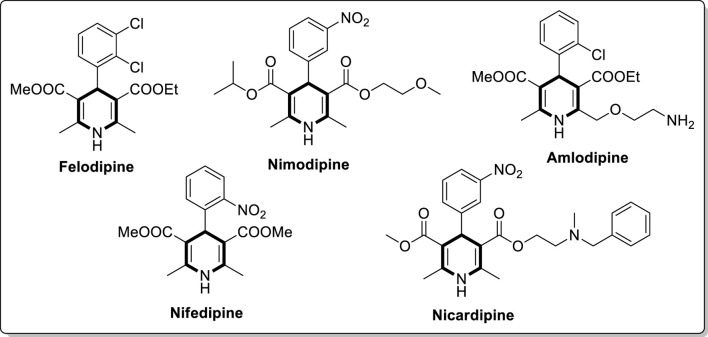
Commercially used 1,4-dihydropyridine drugs.

The 1,4-dihydropyridine is a significant six-membered nitrogen-based heterocyclic molecule, a valuable moiety in medicinal chemistry. In 1881, Arthur R, Hantzsch first reported the dihydropyridine molecule through a one-pot reaction of two moles of ethyl acetoacetate with carbonyl compound and nitrogen source (ammonia or ammonium acetate) (Scheme 1) (Hantzsch, 1881). The process proceeded through the formation of enamine (**I**) and chalcone (**II**) intermediates and further underwent cyclisation to generate a 1,4-dihydropyridine product (Scheme 2) (Katrinsky et al., 1986). Currently, many dihydropyridine drugs are available in the market (felodipine, nimodipine, amlodipine, nifedipine, and nicardipine) (Figure 1). These scaffolds found importance in biological applications, particularly in calcium channel antagonists and treatment of hypertension (Janjua and Mayer, 2003; Triggle, 2003; Edraki et al., 2009). Therefore, the varied bioactive applications of dihydropyridine analogues have considerable attention for synthesising. The current review emphasises the various synthetic approaches for 1,4-dihydropyridine analogues using different metal-based heterogeneous catalyst materials.

### Magnetic Nanocatalysts

Lately, catalytic compounds in synthetic processes have been a topic of concern due to their nature and adverse effects on the environment, drawing attention to eco-friendly alternatives. The emerging challenge was to modify the naturally available compounds for multiple uses without compromising their vitality. The construction of magnetic nanoparticles with promising catalytic activity offers many advantages compared to traditional inorganic catalysts. A huge surface area-to-volume ratio and a high atomic accommodation capacity are advantageous for these particles. These catalysts can be easily separated from the final product using external magnetic fields owing to their magnetic efficiency. Magnetic nanocatalysts have gained paramount importance in organic and industrial reactions because of their interactions with various reagents and environmentally amiable properties, such as anchoring various functional groups or organic moieties onto the hydroxyl projections of their surface area. Integrating transitional metals or other plant-extracted enzymes with Fe_3_O_4_ nanoparticles has evolved as a good strategy. The magnetic nanoparticles coalesce with exchangeable electron ligands such as phosphorous or nitrogen offering the electron-donating sites to encourage affiliation to metal cations to form stable and streamlined catalyst structures. The fabrication of iron-based catalysts has enticed many scientists due to their easy accessibility, high performance, easy separation, and cost-effectiveness. The magnetic nanoparticles were prepared using Fe^+3^ and Fe^+2^ oxides in the ratio of 2:1 co-precipitated in an alkaline environment. The prepared nanoparticles elucidated using various analytical techniques such as FT-IR, SEM, and TEM analysis possess a spherical shape with a diameter of 10–15 nm. Their magnetic saturation was observed through value stream mapping (VSM) data. Catalyst accommodation capacity and microwave array capacity are the two fundamental factors for the optimisation of the catalyst.

**SCHEME 3:**
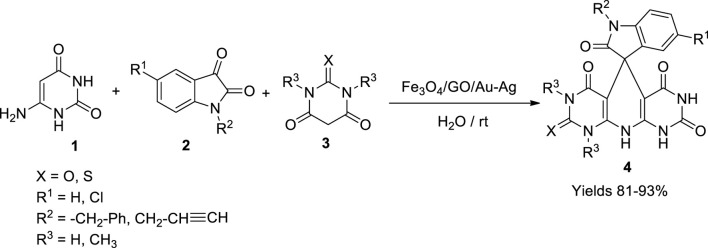
Synthesis of spirooxindole-dihydropyridines by the Fe_3_O_4_/GO/Au-Ag catalyst.

Kavyani *et al.* (Kavyani and Baharfar, 2020) synthesised the (Fe_3_O_4_/GO/Au–Ag) nano-catalyst with Au–Ag particles clipped on Fe_3_O_4_ and graphene oxide. The nanocatalyst’s activity was utilised to synthesise dihydropyridine-spiro oxindole analogues (**4**). The condensation reaction was carried out between uracil-6-amine (**1**), substituted isatin derivatives (**2**), and various barbituric acids (**3**) underwater at room temperature. The nanocatalyst was significantly thermally stable up to 600°C, and the average particle size was about 15 nm. The use of 20 mg of the nanocatalyst gave superior results with high yields (81%–93%) for <5 h of reaction time (Scheme 3). The catalyst was stable up to five successive runs without loss of its catalytic activity. Broad functional group tolerance, excellent yields, green solvent medium, and recyclability are the merits of this protocol.

**SCHEME 4:**
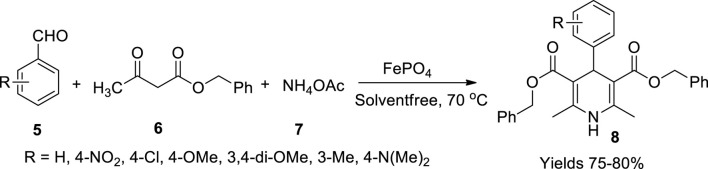
Synthesis of dihydropyridines by the FePO_4_ catalyst.

Behbahani and coworkers (Farahnaz and Banafsheh, 2019) practised the Hantzsch reaction to synthesise 1,4-dihydropyridines (**8**) utilising iron(III) phosphate aiming for a solvent-free synthetic method (Scheme 4). They employed different types of aldehydes (**5**) with benzyl acetoacetate (**6**) and ammonium acetate (**7**) under the influence of a catalyst (Fe_3_PO_4_). The reaction proceeds by activating the carbonyl group of the aldehyde advocated by Fe_3_PO_4_, which acts as a Lewis acid, at a minimal catalytic usage of 10mol% to obtain an appropriate amount of yield. The reaction initially included benzaldehyde, benzyl acetoacetate, and ammonium acetate at an optimal temperature of 70°C. The aldehydes with +I substitution groups like -NO_2_, -OCH_3_, -CH=CH_2_ were observed to serve a better yield of 80% within a reaction time of 50 min using a minimal amount of 5 mol% of the catalyst.

**SCHEME 5:**
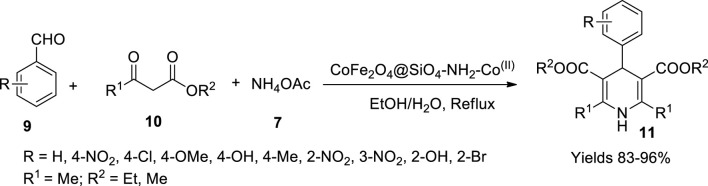
Synthesis of dihydropyridines by the CoFe_2_O_4_@SiO_4_-NH_2_-Co^(II)^ catalyst.

Allahresani *et al.* (Allahresani et al., 2020) synthesised a nanocatalyst with magnetic properties by adhering Co(II) onto CoFe_2_O_4_@SiO_2_ nanoparticles and characterised using various spectroscopic techniques. Catalyst synthesis is a combination of multiple components. The first step involved the preparation of CoFe_2_O_4_ nanoparticles by dissolving Fe (NO_3_)_3_.9H_2_O and Co (NO_3_)_2_.6H_2_O in a molar ratio of 2:1 in 100 ml of water and sonication. A volume of 20 ml of 1 M NaOH solution was added to the resulting solution and subjected to sonication once again. The resulting black precipitate was gathered using an external magnetic field and dried in the oven. The second step consisted of the addition of tetraethylorthosilicate to obtain CoFe_2_O_4_@SiO_2_. In the third step, NH_2_-Pr was synthesised by reacting triethanolamine, triethylenetetramine, and 3-chloropropyltriethoxysilane in ethyl alcohol. Upon adding Co (NO_3_)_2_.6H_2_O to the above-obtained product, NH_2_-Pr-Co(II) formed was dispersed in dry toluene to which a solution of CoFe_2_Co_4_@SiO_2_ in dry toluene was added dropwise, resulting in the precipitate of desired catalyst CoFe_2_O_4_@SiO_2_-NH_2_-Co(II). The XRD values of the synthesised catalyst explored its polycrystalline structure with an average particle size of 20–90 nm, as suggested by TEM analysis. The VSM values indicate that the magnetic properties of the catalyst depended on the surface arrangement of the catalytic particles, which were stable up to 560°C as per thermogravimetric analysis (TGA) analysis. The catalyst (180 mg) facilitated a 96% yield in 2 h, in aqueous ethanol (1:1). Several dihydropyridine derivatives (**11**) have been synthesised in a single-pot mild reaction, including aldehydes (**9**), ethyl acetoacetate (**10**), and ammonium acetate (**7**), aiding CoFe_2_O_4_@SiO_4_-NH_2_-Co(II) as a catalyst owing to its active metallic nucleus, sustainability, and high-yielding capacity (Scheme 5). The reaction was observed to follow the Knoevenagel condensation method and Michael addition to obtain various pyridine derivatives.

**SCHEME 6:**
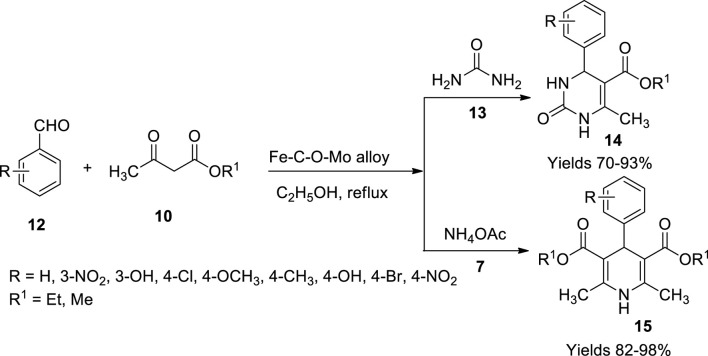
Synthesis of dihydropyrimidinone and dihydropyridines by Fe–C–O–Mo catalyst.

Wua and coworkers (Wu et al., 2020) developed a magnetic nanocatalytic alloy mediated by a wasted screw adopting the top-down method. The catalyst is an alloy combination of iron and molybdenum bridged by carbon and oxygen. The catalyst was prepared by suspending the screws in a solution containing dimethyl sulphoxide and sodium hypochlorite, which was further decomposed by adding 60% nitric acid. The formation of red fuming gas indicates the decomposition initiation due to the expulsion of nitrous acid. The complete dissolution of the screw and the appearance of a brown colour solution marked the completion of the reaction. The required catalyst (Fe–C–O–Mo alloy) was obtained by centrifugation, and the brown-coloured powder was filtered and stored. The performance of the catalyst was examined to prepare dihydropyridines (1**5**) using benzaldehyde (**12**), ethyl acetate (**10**), and ammonium acetate (**7**) as reagents. Dihydropyrimidinone (1**4**) compounds were also reported using urea (**13**) instead of ammonium acetate (**7**) (Scheme 6). The catalyst with stability up to 1,000°C showed worthy competence of 96% yield within a reaction time of 2.5 h with minimal use of 0.1 g of catalyst in the ethyl alcohol medium. Molybdenum acts as a Lewis acid that reacts with the carbonyl group and the beta diketones. Their nucleophilic attack on the reactants yields the desired result.

**SCHEME 7:**
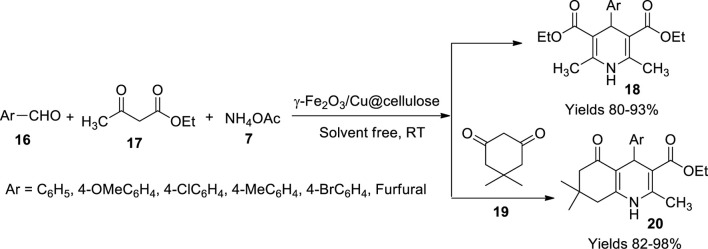
Synthesis of dihydropyridine and hydroquinolines by the Fe_2_O_3_/Cu@cellulose catalyst.

Maleki and his team (Maleki et al., 2019a) have tailored a magnetic bio nanocomposite using copper nanoparticles embedded in a cellulose matrix (γ-Fe_2_O_3_/Cu@cellulose), which showed magnificent catalytic efficiency during the Hantzsch reaction. High yields of 1,4 dihydropyridine (**19**) and polyhydroquinoline (**20**) were observed upon employing γ-Fe_2_O_3_/Cu@cellulose for their preparation. They prepared a homogenous solution of cellulose in an aqueous mixture of sodium hydroxide and urea bearing a ratio of 81:7:21, whose temperature was dropped to −12°C using an ice bath incorporated with salt. An aqueous solution of iron chloride with Fe^+3^ and Fe^+2^ in the ratio of 1:2 was added to the above solution while continuously stirring for 6 h, resulting in γ-Fe_2_O_3_-implanted cellulose assisted by co-precipitation. The CuSO_4_.4H_2_O solution was added to the above mixture; owing to the oxidative nature of the γ-Fe_2_O_3_ nanoparticles, Cu^+2^ is protected from reduction. The mixture was stirred continuously for 2 h before washing and collecting the product. From TEM analysis, the obtained catalyst’s particle size was 25–30 nm, and the EXD data confirmed the presence of C, O, Cu, and Fe. It showed engaging activity with a high yield of around 92%–95% within a short reaction time of 15–20 min in a solvent-free environment (Scheme 7).

**SCHEME 8:**
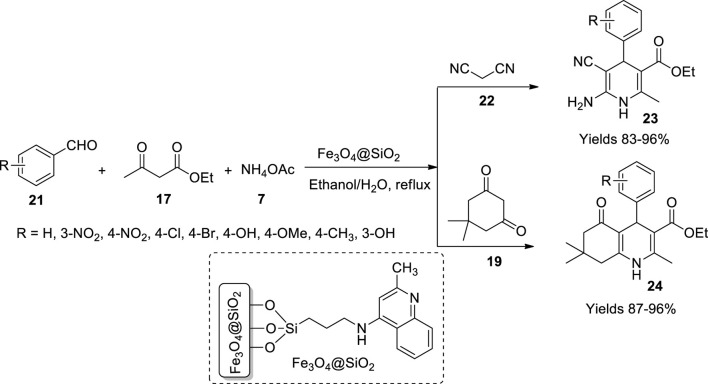
Synthesis of dihydropyridine and hydroquinolines by the Fe_3_O_4_@SiO_2_ catalyst.

Bodaghifard *et al.* (Bodaghifard, 2020) fabricated an organic bolstered magnetic nanocatalyst which was promising in preparing dihydropyridines. The catalyst was manufactured by preparing Fe_3_O_4_ nanoparticles by co-precipitation of Fe^+2^ and Fe^+3^ in a 1:2 ratio under basic pH. The gathered nanoparticles were laminated with silicon using the Stomper method. The obtained Fe_3_O_4_@SiO_2_ nanoparticles were treated with 3-chloropropyltrimethoxysilane resulting in Fe_3_O_4_@SiO_2_-PrCl. 4-Aminoquinaldine adheres to the gathered nanoparticles to bequeath the desired catalyst. The obtained catalyst was subjected to various spectroscopic assays to elucidate its morphology which described spherical nanoparticles with 25–35 nm in diameter, as suggested by SEM. The crystallite size was 20.1 nm as per XRD data. The VSM data advocated the magnetisation of the catalyst to be around 42 emu/g. The efficacy of the material was assessed *via* the synthesis of 1,4-dihydropyridines (**23**) and polyhydroquinoline (**24**) derivatives (Scheme 8). The synthesis process involved benzaldehyde (**21**), ethyl acetoacetate (**17**), malononitrile (**22**), and ammonium acetate (**7**) in equal quantities. The reaction is presumed to follow Knoevenagel condensation to form arylidene malononitrile. The intermediate further participates in Michael addition. The catalyst increased the yield to 96% and dropped the reaction time to 30 min in an aqueous medium. The catalyst material was isolated by applying an external magnetic field.

**SCHEME 9:**
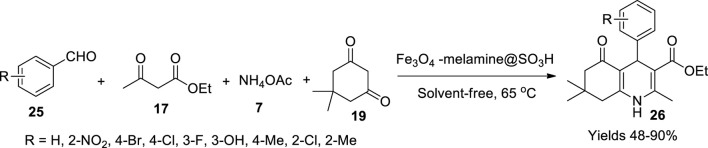
Synthesis of hydroquinolines by the Fe_3_O_4_-melamine@SO_3_H catalyst.

Khazaei *et al.* (Khazaei et al., 2018) worked on high-density ionic liquid that adhered onto magnetic nanoparticle Fe_3_O_4_ and its eminence in the production of hexahydroquinolines. The nanoparticle was initially fabricated using Fe^+2^ and Fe^+3^ in the ratio 1:2 dispersed in water and adding 28% (wt%) of ammonia solution to obtain a black precipitate of Fe_3_O_4_ nanoparticles after continuously stirring the solution at 80°C for 2 h under the influence of the N_2_ environment. A percentage of 28% (wt%) of tetramethyl orthosilicate was added to a solution of Fe_3_O_4_ nanoparticles suspended in ethanol:water (80:20 wt%) mixture to gather Fe_3_O_4_@SiO_2_ nanoparticles. The above-formed nanoparticles were taken in toluene and refluxed with (3-chloropropyl)triethoxysilane to obtain Fe_3_O_4_@SiO_2_@Si-(CH_2_)_3_Cl nanoparticles, which were further added to a solution of melamine and K_2_CO_3_, dissolved in 70 ml of DMSO to affix melamine to the above formed NPs. A methanolic solution of picoline aldehyde was added to the NPs, followed by 30 ml of dichloromethane to the pre-dried NPs under MgSO_4_. A 24-mmol chlorosulphonic solution in 20 ml of dichloromethane was further added dropwise to form Fe_3_O_4_@SiO_2_@Si-(CH_2_)_3_@melamine-picolineimine@SO_3_H nanoparticles. Different spectral data obtained from FT-IR and XRD suggest that the crystallite size of the catalyst is 14.98 nm whereas SEM analysis advocates for the high interaction of the HSO_3_ group on the surface along with TEM data which proves the lamination of the core–shell. The EDS analysis helped elucidate the nanoparticles’ morphology with dominant levels of Fe, Si, O, N, S, and Cl. The VSM analysis evaluated its magnetic nature, and the material was stable at 600°C as suggested by TGA data. A series of polyhydroquinolines (**26**) were reported using aryl aldehydes (**25**), β-keto esters (**17**), dimedones (**19**), and ammonium acetate (**7**) in equal proportions confirming the catalytic potential of the magnetic nanoparticles (Scheme 9). The obtained products were characterised using FT-IR, ^1^H, and ^13^C NMR while being evaluated by TLC. The highest yields were observed when the aryl aldehyde had an electron-donating group of +I category. With p-anisaldehyde, a 90% yield was registered under solvent-free abode with 40 mg of the catalyst at 65°C. The catalyst sustained its viability for four cycles.

**SCHEME 10:**
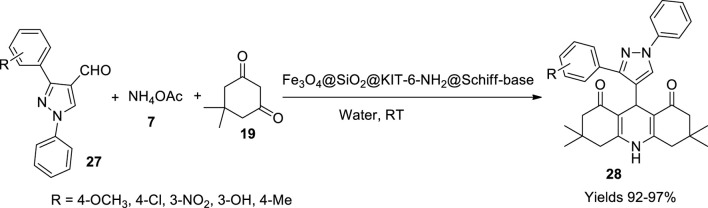
Synthesis of hydroquinolines by the Fe_3_O_4_-SiO_2_ Schiff–base catalyst.

Fekri *et al.* (Fekri et al., 2020) have fabricated a Copper/Schiff–base complex immobilised on KIT-6-NH_2_ nanoparticles, which can be used as a catalyst to prepare dihydropyridine derivatives. Several steps were followed to tailor the desired catalyst complex. Initially, Fe_3_O_4_ nanoparticles were synthesised using a mixture of Fe^+2^ and Fe^+3^ salts in the ratio of 1:2 treated with ammonia solution to obtain a black precipitate of nanoparticles. The nanoparticles were laminated with a layer of silica and further overlapped with Kit-6 silica to impart a mesoporous nature to the product. The post-synthetic grafting method was used for the amine functionalisation of the magnetic nanoparticles, where 3-aminopropyl triethoxysilane was silylated using surface silanol. The Schiff’s base was adhered onto the amino functionalised magnetic nanoparticles (MNPs) using 1-(2-hydroxy-5-(4-nitrophenyl)-diazenyl)-phenyl)-ethenone. The final catalytic conclusion involves the induction of copper to the MNPs using CuCl_2_ to achieve a Copper/Schiff–base complex immobilised on KIT-6-NH_2_ magnetic nanoparticles. The gathered compound was morphologically elucidated using various methods such as BET analysis, representing its particle size of 51.43°m^2^/g. The VSM is used to assess its magnetic nature. TEM images explored the spherical shape of the particles with a diameter of 33–40 nm along with aggregation and stacking of particles. XRD showed a highly crystalline cubic spinel structure, and the elemental analysis energy-dispersive X-ray analysis (EDX) revealed that the catalyst contains Fe, O, N, C, Cu, and Si. TGA analysis suggested stability up to 520°C. The catalytic activity of MNPs was tested on Hantszch’s single-pot reaction method to synthesise polyhydroquinolines (**28**) using aryl aldehyde (**27**), cyclic ketone (**19**), and ammonium acetate (**7**) in the presence of the above-prepared catalyst (Scheme 10). The reaction was assumed to follow Knoevenagel condensation followed by Michael addition and intramolecular addition. The reaction conclusion was determined using TCL, where the products were characterised using FT-IR, ^1^H, and ^13^C NMR methods. The catalyst proved to enhance the reaction with high yields around 92%–97% within 15 min of reaction time. The catalyst was separated magnetically and used without any resentment for seven consecutive cycles.

**SCHEME 11:**
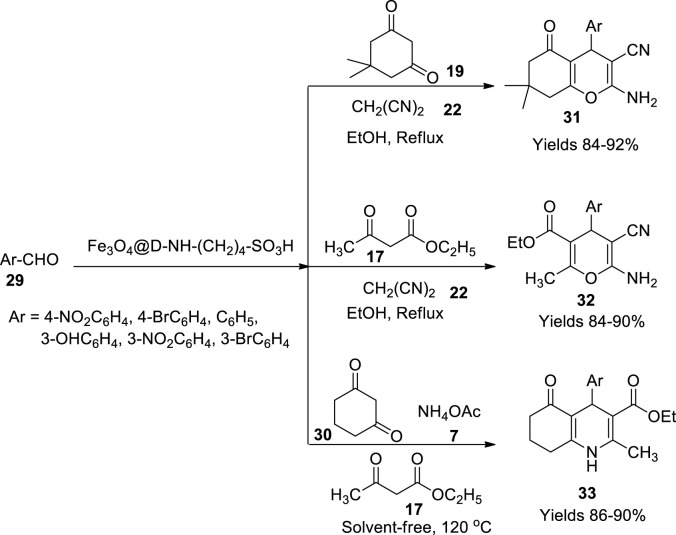
Synthesis of hydroquinolines by the Fe_3_O_4_ catalyst.

Maleki and his colleagues (Maleki et al., 2019b) fabricated a heterogeneous magnetic nanocatalyst bolstered with sulphonyl skeleton to prepare pyrans and polyhydroquinolines. Initially, 1 g of diethylenetriamine in 5 ml of dried methane and 10 g methyl acrylate in 25 ml of dried methanol were stirred continuously till the solvent was evaporated entirely to obtain pioneer dendrimer. The dendrimer terminals with 5-OCH_3_ groups were modified to 5-NH_2_ by dissolving it in a solution of 20 ml methanol and 40 ml of ethylenediamine. The modified dendrite generation was isolated from unreacted reagent and methanol using vacuum. The acquired dendrite was further modified by adding 30 ml of methyl acrylate to 6.74 g of the modified pioneer dendrite. The stirring was continued for 7 days in an inert environment to gain a thick yellow liquid consisting of 10 branched dendrimers with -OCH_3_, which was further modified using 60 ml of ethylenediamine, and the procedure was repeated to gather a pale-yellow oily material. The prepared dendritic material was magnetised by dispersing it in a 1:2 ratio Fe^+2^ and Fe^+3^ solution and subjecting it to sonication. It was later functionalised by adding 2.3 ml of 1,4-butane sultone dropwise followed by sonication. The final Fe_3_O_4_@D-NH-(CH_2_)_4_-SO_3_H nanoparticles were isolated using an external magnetic field. The prepared catalyst was evaluated using SEM and TEM imaging, representing the uniform distribution of nanoparticles with spherical structure. The catalyst was stable up to 700°C owing to the dendrimers of D-NH-(CH_2_)_4_-SO_3_H side chains. The prepared catalyst was used in the production of tetrahydrobenzo pyrans (**31**), 2-amino-3-cyano-4H-pyrans (**32**), and polyhydroquinolines (**33**). The former pyrans were prepared by refluxing a reaction mixture of aldehydes (**29**), malononitrile (**22**), and 1,3-cyclohexanedione (**19**) or ethyl acetoacetate (**17**) in 5 ml of ethanol in the presence of the above-prepared catalyst. The polyhydroquinolines were produced by refluxing a reaction mixture containing aldehydes (**29**), 1,3-cyclohexanedione (**19**), ethyl acetoacetate (**17**), and ammonium acetate (**7**) in the presence of the same catalyst. The reaction was assumed to proceed through Knoevenagel condensation to produce 2-benzylidenemelanonitrile. The intermediate endures Michael with enolised dimedone to generate enamine intermediate. The enamine undergoes cyclisation and tautomerism to give the product a high yield (96%) within 20 min using 0.05 g of the catalyst (Scheme 11). The gathered products were subjected to FT-IR, ^1^H, and ^13^C NMR spectroscopy.

**SCHEME 12:**
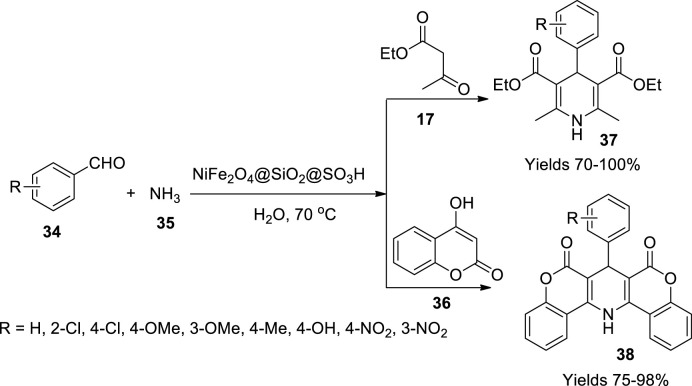
Synthesis of dihydropyridines by the NiFe_2_O_4_-SiO_2_@SO_3_H catalyst.

Zeynizadeh *et al.* (Zeynizadeh et al., 2019) fabricated a catalyst for the synthesis of 1,4-dihydropyridines by adhering sulphonic acid onto silica-layered NiFe_2_O_4_, which could be isolated magnetically and reused multiple times. Initially, the magnetic nanoparticle base was produced by grinding a reaction mixture containing nickel acetate, ferric nitrate, sodium hydroxide, and sodium chloride taken in the ratio of 1:2:8:2. The ground mixture was washed with DM water and dried at 80°C for 2 h and further calcinated at 700°C–900°C for 2 h. The prepared nanocatalyst was functionalised with a silica solution. A volume of 1.5 g of the prepared nanoparticles along with 200 ml of *i*-PrOH and 20 ml of DM water was irradiated with ultrasound and added to a solution containing 5.36 g of PEG-400 in 20 ml DM water, 10 ml of 28% aqueous ammonia, and 2 ml of tetraethyl orthosilicate (TEOS) and stirred continuously for a day and a half to obtain NiFe_2_O_4_@SiO_2_ nanoparticles. A volume of 2 g of the prepared nanoparticles was suspended in 15 ml of chloroform to which 0.5 g of sulphonyl chloride was added dropwise and stirred while maintaining a temperature range ≥5°C. The resulting mixture was filtered and dried at room temperature to obtain a black precipitate of NiFe_2_O_4_@SiO_2_@SO_3_H nanoparticles. The gathered catalytic nanoparticles were elucidated using various techniques such as XRD analysis which describes its rhombic crystalline structure. The SEM analysis showed a 60–90-nm nanosize, and the material was stable up to 600°C. The gathered catalyst was subjected to trial to determine its efficiency for the preparation of 1,4-dihydropyridines (**37** and **38**) by employing a single-pot Hantzsch reaction mixture consisting of aromatic aldehyde (**34**), ethyl acetoacetate (**17**), or 4-hydroxycoumarin (**36**) and 28% aqueous ammonia 28% (**35**) in 2 ml of DM water (Scheme 12). The completion of the reaction was determined using TLC using n-hexane and ethyl acetate as eluent. The resulting product was isolated using the solvent extraction method and dried over Na_2_SO_4_ under reduced pressure. The prepared 1,4-dihydropyridine derivatives were characterised using FT-IR, ^1^H, and ^13^C NMR spectroscopy. The products were with a high yield of 95% within a reaction time of 20 min with the catalyst sustainability for seven consecutive cycles. Besides the synthesis of dihydropyridines, Cu–adenine–boehmite was also used in the Suzuki coupling reaction and oxidation of sulphides.

**SCHEME 13:**
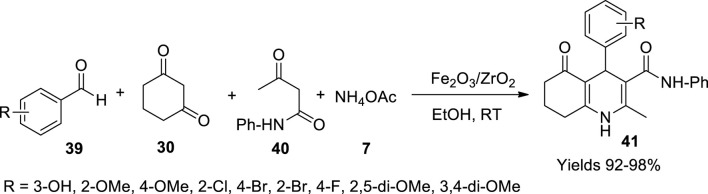
Synthesis of hydroquinolines by the Fe_2_O_3_/ZrO_2_ catalyst.

Bhaskaruni *et al.* (Bhaskaruni et al., 2019) have constructed a green route to prepare 1,4-dihydropyridines under the catalytic influence of Fe_2_O_3_/ZrO_2_. The catalyst was prepared by wet impregnating 2 g of zirconium oxide with equal amounts of ferric nitrate in 50 ml of water while continuously stirring at room temperature for 7 h. The obtained slurry was filtered and vacuum dried at 120°C for 5 h followed by calcinating it at 450°C for 4 h. The prepared material was elucidated using various techniques, which described its polycrystalline structure as having a 10.23-nm diameter. The nanoparticles were irregular oval-shaped with a particle size of 8.5 nm as represented by TEM and SEM analysis. The surface area of the catalyst was 84.34 cm^2^/g with a mesoporous cavity size of 8.9 nm and a volume of 0.25 cm^3^/g, through BET data. The catalytic activity of Fe_2_O_3_/ZrO_2_ was assessed in preparing hydroquinolines (**41**). A single-pot system consisting of equimolar amounts of aromatic aldehyde (**39**), 1,3-cyclohexadiene (**30**), acetoacetanilide (**40**), and ammonium acetate (**7**) was subjected to stirring for 20 min at room temperature (Scheme 13). The catalyst was isolated from the products through filtration. The product was extracted using ethyl acetate and recrystallised from ethanol. The reaction is presumed to follow Knoevenagel condensation and Michael addition to form an enamine which further undergoes tautomerism. An excellent 98% yield was gained with minimal use of the catalyst within 20 min. The gathered product was confirmed using various spectroscopic techniques.

**SCHEME 14:**
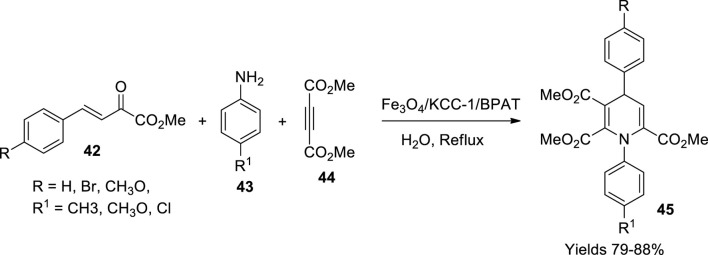
Synthesis of dihydropyridines by the Fe_3_O_4_/KCC-1 catalyst.

Sadeghzadeh reported a magnetic KCC1 catalyst stabilised by bis(4-pyridylamino)triazine, a green catalyst with good chemo selectivity (Sadeghzadeh, 2016). The production of Fe_3_O_4_ nanoparticles was achieved by dissolving Fe^+2^ and Fe^+3^ in 200 ml of water with a ratio of 1:3 and adding 2 ml of concentrated ammonium solution along with 0.2 g of tetraethyl orthosilicate and continuously stirring for a day. The nanoparticles of Fe_3_O_4_/SiO_2_ were gathered by filtering the obtained black precipitate and air drying it at 60°C after washing. A volume of 0.25 g of the gathered nanoparticles was dispersed in 30 ml of urea solution containing 0.3 g of urea and subjected to sonication for an hour. To 0.5 g of acetylpyridine bromide dissolved in a mixture of 0.75 ml of n-pentanol and 30 ml of cyclohexane, 1.25 g of tetraethyl orthosilicate was slowly added under continuous stirring at 120°C for 5 h. The desired microspheres were isolated using an external magnetic field and washed in acetone and oven-dried at 40°C overnight followed by calcination at 550°C for 5 h. A volume of 1 g of the gathered nanoparticles along with 20 mmol of sodium hydride was dispersed in 20 ml of tetrahydrofuran (THF) and subjected to ultrasonication which was added with 22 mmol of 3-aminopropyltriethoxysilane while continuously stirring for 6 h at 60°C the resulting Fe_3_O_4_/SiO_2_/KCC-1/aminopropyl MNPs, which were washed and vacuum dried for another 2 h at 60°C. A volume of 1 g of the gathered product was added to a solution of 1 ml of N-ethyldiisopropylamine and 10 ml of dry THF while maintaining the temperature below 5°C and adding 1 g of triazine trichloride while continuously stirring for a whole day. Fe_3_O_4_/KCC-1/TCl_2_ nanoparticles were gathered and washed with hot toluene to isolate pure products. A volume of 1 g of the currently gathered nanoparticles along with 1 g of pyridine was dissolved in a solution of 1.3 ml of N-ethyl diisopropylamine and 10 ml of dry toluene while continuously stirring for 16 h at 80°C. Fe_3_O_4_/KCC-1/BPAT nanoparticles were separated from the reaction solution by an external magnet, washed with ethanol, and dried at 50°C. Represented by SEM and TEM data, the stoked nanoparticles had 20–30-nm particle size. The magnetic saturation was 55.1 and 42.4 emu/g following VSM data, and the material was stable up to 700°C. The catalytic efficiency of the prepared nanocomposite was assessed by a single-pot synthesis of 1,4-dihydropyridines (**45**). The equimolar mixture of primary amine (**43**), dimethyl acetylene dicarboxylate (**44**), and methyl (arylmethylide) pyruvate (**42**) was refluxed for 4 h in the presence of Fe_3_O_4_/KCC-1/BPAT (Scheme 14). The solvent was removed by dropping its pressure to obtain an oily residue, treated with methanol, and recrystallised to isolate pure product. The reaction proceeds through Knoevenagel condensation along with Michael addition. Various spectroscopic methods characterised the isolated 1,4-dihydropyridine derivative. A good yield of around 85% with 8 mg of the catalyst within a reaction time of 2 h was reported.

**SCHEME 15:**
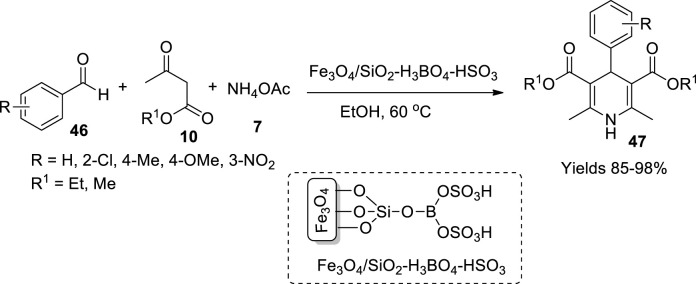
Synthesis of dihydropyridines by the Fe_3_O_4_/SiO_2_ catalyst.

Azizi and his colleagues (Heydari et al., 2016) have fabricated sulphated boric acid-functionalised magnetic nanoparticles and utilised them as a catalyst to synthesise esters from the Hantzsch reaction method. The magnetic nanoparticles were synthesised by refluxing a reaction mixture of Fe^+2^ and Fe^+3^ with a molar ratio of 1:2 and further treated with chlorosulphonic acid. The functionalised nanoparticles were reacted with boric acid, adhered to silica to obtain Fe_3_O_4_/SiO_2_-H_3_BO_4_-HSO_3_ nanoparticles, and filtered and washed with ethanol to isolate pure material. XRD analysis suggests a perovskite crystalline structure and stability up to 350°C as represented by TGA data with a boric acid concentration of 1.2 mmol/g. The catalytic efficiency was assessed in the Hantzsch ester formation reaction to synthesise 1.4-dihydropyridine derivatives (**47**). The single-pot reaction involved equimolar amounts of aldehyde (**46**) and ammonium acetate (**7**) and a double equimolar quantity of β-keto esters (**10**) (Scheme 15). The reaction proceeds through Knoevenagel condensation followed by Michael addition. The obtained precipitate was filtered and dried to gather the desired derivatives and characterised using various spectroscopic methods. The catalyst was isolated from the reaction mixture by an external magnetic field and reused for four to five reaction cycles without losing its efficiency.

**SCHEME 16:**
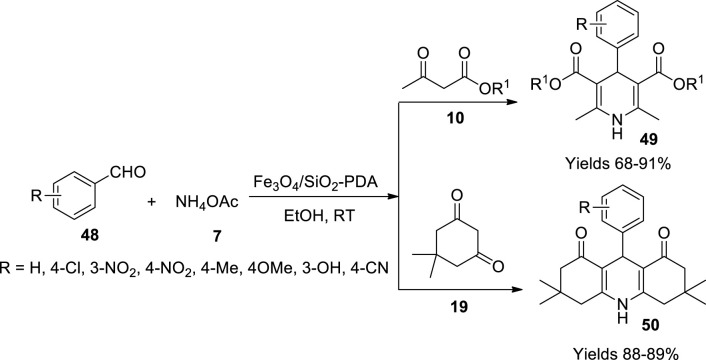
Synthesis of dihydropyridine and hydroquinolines by the Fe_3_O_4_/SiO_2_-PDA catalyst.

Taheri-Ledari *et al.* (Taheri-Ledari et al., 2019) have investigated a comparative synthetic method for preparing pyrimidine-2,4-diamine-functionalised magnetic nanoparticles *via* refluxing, ultrasonication, and microwave methods to prepare 1,4-dihydropyridines. The Fe_3_O_4_ nanoparticles initially prepared were laminated with silica in the presence of PEG-300 and aqueous ammonia using TEOS as a silica source. The nanoparticles were manipulated with TMVS to adhere to vinyl terminals and were sonicated after adding 50 ppm melamine solution and then transferred into an ultrasound bath 50 KHz at 50°C. The obtained nanoparticles were separated using an external magnetic field, washed with ethanol, and then dried at 60°C. The gathered nanocomposites were characterised by FT-IR, ^1^H NMR, ^13^C NMR, mass, XRD, TGA, SEM, and AFM analyses. The analyses advocate enhanced texture conservation surface functionalisation along with reduced aggregation of the nanoparticles. High yielding capacity with 89% product formation dihydropyridines (**49**) and hydroquinolines (**50**) was observed within a short reaction time of 10 min (Scheme 16).

### Silica-Based Catalysts

Silica has been used as a bolster for other adhesive elements to construct an efficient catalytic structure. Owing to its colossal surface area of 800 m^2^/g, it can accommodate many reagent particles on its surface, exposing them to interact with one another to obtain the desired product. It is a readily available material that can be restructured with acidic functional groups such as phenolic or carboxylic groups or other cationic moieties. The silica-based nanostructures were fabricated to serve as efficient, recyclable material with intransigent sustainability. Usually, silica is doped with different d-block elements to enhance catalytic efficiency by offering amplification of reagent interaction. Various methods such as co-precipitation, sol–gel, micro-emulsion, laser pyrolysis, thermal decomposition, hydrothermal process, sonication, and microwave irradiation have been utilised (Neamani et al., 2020).

**SCHEME 17:**
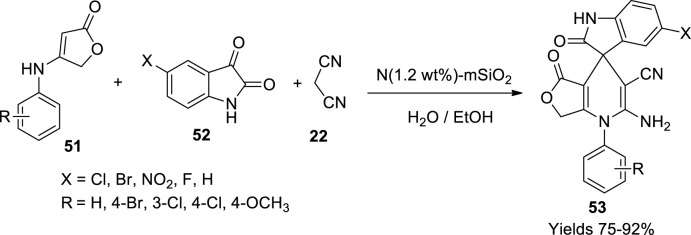
Synthesis of spiro oxindole-1,4-dihydropyridines by the N-doped silica catalyst.

Neamani and his colleagues (Neamani et al., 2020) developed basic hollow mesoporous N-doped silica rods with magnetic properties. The material synthesis consisted of several steps where a mixture of multilayered carbon nanotubes was dissolved in methylene chloride and then subjected to sonication. A mix of acetic acid and KMnO_4_ was added and agitated at 80°C to ensure hydroxylation of multiwalled carbon nanotubes. An Fe^+3^ and Fe^+2^ solution in 1.75 ratios was then added, and the product was washed with ammonia. The magnetised nanotubes were then doped with silica using a TEOS and DEA solution mixture while using CTAB as a structural directing agent. The vast surface area of around 10.4 and 493.8 m^3^/g was established through BET analysis. Spiro oxindole-1,4-dihydropyridine derivatives (**53**) were synthesised in the presence of the prepared material as a catalyst. A good 80% yield was observed with minimal use of N-1.2% weight of m-SiO_2_ in the presence of ethyl alcohol. The sequential mechanism of Knoevenagel condensation followed by Michael addition and cyclisation was observed. +I substituents in the reactant heterocyclic compound aided better yield as high as 92% within 6 h (Scheme 17). Also, the catalyst showed a good deal of consistency after several consequent usages.

**SCHEME 18:**
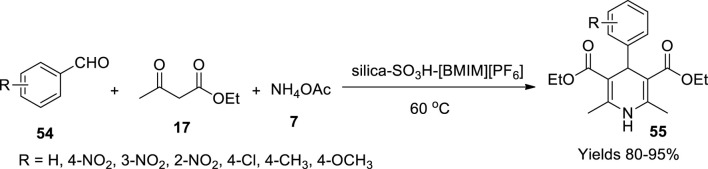
Synthesis of dihydropyridines by the silica-SO_3_H-[BMIM][PF_6_] catalyst.

Sharma and Gupta have composed a silica-based sulphonic acid catalyst that was laminated with ionic liquid (Sharma and Gupta, 2015). The silica activated with 50% HCl (HCl: H_2_O 1:1) was treated with 3-mercaptopropyl (trimethoxyl) silica and toluene and refluxed for 24 h. The resulting 3-mercaptopropyl silica is then filtered and treated with 30% H_2_O_2_ solution and concentrated H_2_SO_4_ while constantly stirring for 20 h. The obtained material by filtration was dried and characterised. The catalyst was a porous powder with an amorphous structure of the particles as deemed by SEM and TEM imaging. The material retained its form up to 360°C. A [BMIM][PF_6_]-laminated (20%) silica-based sulphonic acid catalyst was found to be most efficient among other derivatives. The material showed excellent catalytic activity in synthesising 1,4-dihydropyridines (**55**), offering high yields of 95% within a reaction time of 15 min (Scheme 18). The catalyst showed similar activity even after several cycles.

**SCHEME 19:**
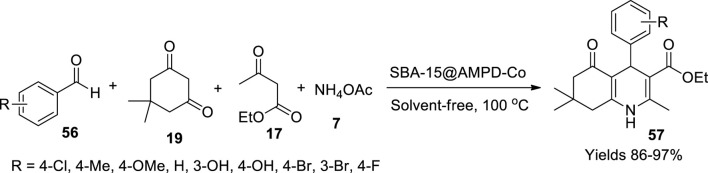
Synthesis of hydroquinolines by the SBA-15@AMPD-Co catalyst.

Choghamarani and his team (Ghorbani-Choghamarani et al., 2019) have fabricated a green heterogeneous catalyst by adhering cobalt onto the surface of SBA-15, which showed exceptional ability in oxidising sulphides and the manufacture of polyhydroquinolines. The mesoporous SBA-15 was prepared by the sol–gel method and functionalised by refluxing it with 1.5 ml of 3-chloropropyltrimethoxysilane in 20 ml of dry toluene medium. The resulting CPTMS (SBA-15-Cl) was functionalised by refluxing it with 2-amino-2-methyl-1,3-propanediol and triethylamine dispersed in dry toluene. The final product was adhered to with cobalt by using 0.73 g of Co(NO_3_)_2_.6H_2_O as the cobalt source and refluxing it for 16 h, which was later washed with ethanol to obtain the pure product free from by-products. The homogenous dispersion of the Co metal onto the surface of the modified SBA-15 gave a mesoporous cavity with a volume of 0.534 cm^3^/g and a surface area of 238.47 m^2^/g, which was represented by X-ray mapping and BET analysis, respectively. The prepared catalyst was used to synthesise polyhydroquinolines using the Hantzsch reaction mixture and oxidation of sulphides. The polyhydroquinolines (**57**) were prepared by using a reaction mixture of dimedone (**19**), aldehyde (**56**), ammonium acetate (**7**), and ethyl acetoacetate (**17**) using 1 mmol each aided with 8 g of SBA-15@AMPD-Co nanocatalyst (Scheme 19). The prepared yellow solid was filtered and washed with ethanol to gather polyhydroquinolines in the range of 86%–97% within 35 min. Apart from the above method, sulphides were oxidised using SBA-15@AMPD-Co in the presence of H_2_O_2_ in a solvent-free environment. TLC determined the reaction completion, and the gathered products were confirmed using FT-IR, ^1^H, and ^13^C NMR spectroscopic data.

### Zirconium-Based Catalysts

Zirconium oxide or zirconia is a ceramic oxide material used as a catalyst by incorporating it in various polymeric structures, making it a biocompatible catalyst. Due to its high thermal capacity and thermal insulation properties, zirconia can be used at high temperatures where other similar materials fail. According to the literature and structural survey, zirconia acts as a Lewis acid and base and thus as a bifunctional catalyst. The optimisation of zirconia is still a work in progress because of its exiguous charge transfer and pH sensitivity. Zirconia is also used with other transitional metals such as silver, palladium, and iron or other metallic oxides such as ceria, titania, silica, and vanadia. Zirconia is used as a catalyst in many organic transformations and shift reactions such as epoxidation, isomerisation, and alkylations. Synthetic methods such as co-precipitation, which is used as a wet process, and the sol–gel method and hydrothermal processes aid in preparing solid zirconia particles. Its physical characteristics have been studied intensively to analyse its surface area, nanoparticle structure, and size using various analytical spectroscopic techniques such as FT-IR, SEM, and TEM analysis.

Majhi and his team (Majhi et al., 2019) worked on fabricating a microporous heterogeneous catalyst Al-pillared α-ZrP adhered with sulphamic acid to be employed during the preparation of 1,4-dihydropyridines. The tailoring protocol involves a set of subsequent steps initiated with the preparation of α-zirconium phosphate (ZP) by dissolving 10 g of ZrOCl_2_.8H_2_O in 100 ml of phosphoric acid in a round-bottomed flask equipped with a reflux condenser and refluxed for 24 h. The solid formed as a result is isolated *via* centrifugation and collected by decanting the supernatant. The gathered solid was washed with hot DM water to get rid of any unwanted reagents like AgCl. The accumulated product was dried in a hot air oven to obtain α-ZrP compound.

**SCHEME 20:**
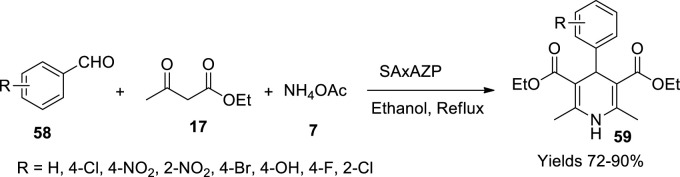
Synthesis of dihydropyridines by the zirconium phosphate catalyst.

Further, 2 g of the earlier prepared compound was dissolved in 200 ml of 0.1 M n-propyl amine solution while continuously stirring for 24 h to form an opaque white colloidal solution subjected to *in-situ* polymerisation to obtain alumina pillared α-ZrP (AZP). Alternatively, 250 ml of 0.2 M AlCl_3_ solution was added to the colloidal solution with continuous stirring to which 1.2 M NaOH solution was introduced in a molar ratio of 1:2 with Al. The solid obtained is centrifuged to collect it and washed with hot DM water to eliminate unwanted Cl^−^ molecules and dried at 100°C. The gathered product was ground to powder and subjected to calcination for 2 h at 300°C to achieve AZP. Furthermore, 1 g of AZP was dissolved in a 20-ml solution of SA in water to gain a mesoporous surface. The aqueous solution was continuously stirred for 6 h at 90°C to attain a solid and further dried at 120°C to achieve a SAxAZP composite. The catalyst was subjected to various elucidation techniques, including XRD, which exposed its crystalline structure with an H3-type surface area of 118 m^2^/g and a pore volume of 0.19 cm^3^/g with a particle size of 25–50 nm as represented by SEM analysis. TGA analysis showed that the catalyst was stable up to 600°C. Its catalytic activity was evaluated using the Hantzsch reaction to prepare 1,4-dihydropyridines (**59**), exercising single-pot synthesis with a reaction mixture of aryl aldehyde (**58**), acetoacetic esters (**17**), and ammonium acetate (**7**) in the presence of the above-obtained catalyst (Scheme 20). The reaction proceeded through Knoevenagel condensation and Michael addition with enhanced Bronsted acid sites. The yield obtained ranged from 75% to 90% depending on the aryl aldehyde used in ethyl alcohol. The products gained were characterised using FT-IR, ^1^H, and ^13^C NMR methods.

**SCHEME 21:**
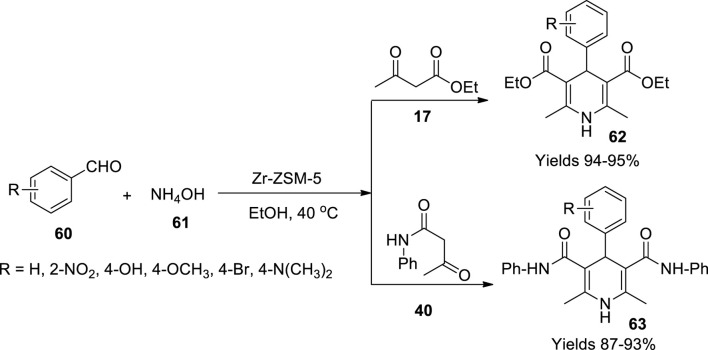
Synthesis of dihydropyridines by th eZr-ZSM-5 catalyst.

Kusampally *et al.* (Kusampally et al., 2020) studied diverse reaction conditions to synthesise 1.4-dihydropyridines (**62** and **63**) using zeolite-anchored Zr-ZSM-5 as an illustrious catalyst. To tailor the material, ammonium ZSM-5 was initially calcinated using a muffled furnace set to 500°C under aerobic conditions subjected to a heating ramp rate of 5°C per minute for 4 h to acquire H-ZSM-5. A solution of zirconium nitrate in water was added dropwise into H-ZSM-5 while constantly stirring the solution to incorporate zirconium nitrate into H-ZSM-5. The generated catalyst was dried at 120°C for 12 h, and the dry product was recalcinated in a muffled furnace at 500°C. The gathered catalyst was subjected to XRD, SEM/EDS, and BET SA-PSD spectroscopic techniques, exploring its crystalline structure. The high surface area of 341 m^2^/g allows the zeolite catalyst to interact with the reagents increasing the active sites. The classic Hantzsch reaction was examined using the Zr-ZSM-5 Zeolite catalyst in a single-pot reaction. The process involved benzaldehyde (**60**), ethyl acetoacetate (**17**), and ammonium hydroxide (**61**) in obtaining 1,4-dihydropyridines (Scheme 21). The use of catalyst gained a high yield of the product, around 96%, with minimal use of 30 mg within 25–35 min. With the microwave-assisted protocol, the reaction mixture and catalyst were placed in a preheated microwave oven at a temperature of 300°C. The enamine formed as an intermediate participated in Michael addition to give the target compound. The catalyst retained its viability even after five consequent cycles. Apart from its sustainability, the catalyst’s efficiency to reduce the reminiscent reagent brands it an eco-catalyst.

**SCHEME 22:**
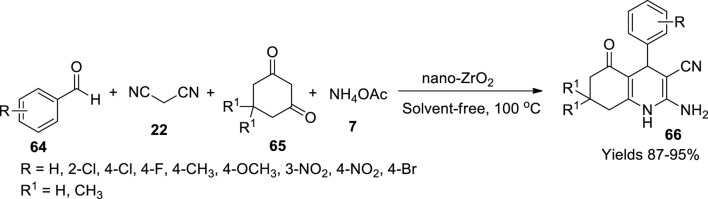
Synthesis of hydroquinolines by the nano-ZrO_2_ catalyst.

Amoozadeh and his colleagues (Amoozadeh et al., 2016) have fabricated a solid heterogeneous catalyst, the sulphonic acid immobilised on nano zirconia. Initially, nano-ZrO_2_ particles were produced *via* chemical precipitation of a 100-ml solution of ZrOCl_2_.8H_2_O, whose pH was adjusted to 10 by adding 2 M NaOH solution. The precipitate was filtered and dried at 120°C, which was later calcinated over a temperature range of 500–1,200°C. Later 0.5 ml of chlorosulphonic acid was introduced into nano-ZrO_2_ dispersed in dry dichloromethane. The obtained reaction mixture was transferred into a suction flask facilitated with a constant-pressure dropping funnel and a gas inlet tube, allowing the conduction of HCl gas to be adsorbed onto the solvent, i.e., water, while continuously stirring the solution. A light cream colour powder was isolated after 30 min, which was washed with ethanol and dried at 100°C. It was found stable up to 150°C. FE-SEM data established the spherical shape of 30–40 nm in diameter. Its crystalline structure through XRD analysis showed a growth rate of 554. In a single-pot preparation of polyhydroquinoline derivatives in a solvent-free environment, the catalyst exhibited good performance. A reaction mixture consisting of 1,3-cyclic diketone (**65**), different aromatic aldehydes (**64**), ammonium acetate (**7**), and malononitrile (**22**) was used to produce polyhydroquinoline derivatives (**66**) (Scheme 22). The obtained products were filtered and washed with hot ethanol to eliminate any unreacted reagents and dried at optimum temperatures. The gathered hydroxyquinoline derivatives were structurally elucidated using FT-IR, ^1^H, and ^13^C NMR spectroscopic analysis; high yields of products ranging from 87% to 95% were obtained advocating its catalytic potential. Optimal conditions for the preparation were determined through the central composite deposit.

**SCHEME 23:**
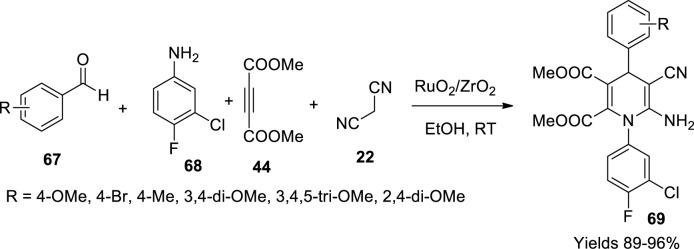
Synthesis of dihydropyridines by the RuO_2_/ZrO_2_ catalyst.

Bhaskaruni *et al.* (Bhaskaruni et al., 2017) have constructed a heterogeneous catalyst, RuO_2_/ZrO_2_, for the preparation of dihydropyridine derivatives (**69**), adopting a single-pot synthetic method using a reaction mixture consisting of equimolar amounts of aldehyde (**67**), malononitrile (**22**), diethyl acetylene dicarboxylate (**44**), and 3-chloro 4-fluoroaniline (**68**) under the influence of RuO_2_/ZrO_2_ in 10 ml of ethyl alcohol medium. The reaction mixture was continuously stirred, and the produced derivatives were filtered and washed with ethanol (Scheme 23). The gathered derivatives were spectroscopically analysed using FTIR, ^1^H, and ^13^C NMR techniques. The catalyst was prepared through the wet impregnation technique by dissolving equal amounts of ZrO_2_ and RuCl_3_.xH_2_O in 100 ml of water while continuously stirring at room temperature for 8 h. The slurry obtained from filtration was oven-dried for 5 h at 110°C–120°C followed by calcination at 450°C for another 5 h to gain the Ru_2_O/ZrO_2_ whose structure was explored using various analytical techniques such as XRD analysis describing its polycrystalline structure with a diameter of 6.3 nm while the particle size of the nanoparticles was 11 nm with irregular shapes with a uniform dispersion of RuO_2_ onto the zirconium surface as assessed through TEM and SEM data. The BET analysis of the catalyst exposed its mesoporous surface area of 41.99 m^2^/g with a pore volume of 0.134 cm^3^/g and a pore size of 12.7 nm. It was observed to retain its catalytic viability for seven consecutive cycles without losing its efficiency.

**SCHEME 24:**
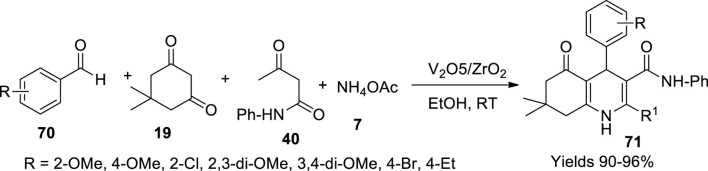
Synthesis of hydroquinolines by the V_2_O_5_/ZrO_2_ catalyst.

Bhaskaruni *et al.* (Bhaskaruni et al., 2018) have constructed a contemporary reaction method under the catalytic influence of V_2_O_5_/ZrO_2_ adopting a single-pot cyclic condensation of a reaction mixture consisting of equimolar amounts of benzaldehyde (**70**), 5,5-dimethyl-1,3-cyclohexanedione (**19**), acetoacetanilide (**40**), and ammonium acetate (**7**) in the presence of V_2_O_5_/ZrO_2_ nanoparticles using 5 ml of ethanol as solvent (Scheme 24). The reaction mixture is continuously stirred for 15 min, and then the desired hydroxyquinoline products (**71**) were isolated through solvent extraction using ethyl acetate followed by evaporation. The reaction proceeded through Knoevenagel condensation along with Michael addition and cyclisation. The gathered product was recrystallised from ethanol to gain a pure product which was subjected to various spectroscopic analyses for characterisation. The catalyst was prepared by wet impregnation method by dissolving equal amounts of V_2_O_5_ and ZrO_2_ in 100–150 ml of DM water while continuously stirring at room temperature for 8 h. The slurry obtained from filtration was oven-dried for 5 h at 120°C–150°C followed by calcination at 450°C for another 5 h to gain V_2_O_5_/ZrO_2_. Its structure explored using various analytical techniques such as XRD analysis confirms its polycrystalline structure with a 7.6 nm diameter. The SEM imaging and TEM imaging display massive irregular structures due to the aggregation of zirconium and vanadium moieties. The surface area of the catalyst was 89.2219 m^2^/g with a mesoporous pore volume of 0.327 cm^3^/g and a pore size of 10.32 nm. The catalytic use gained high yields around 90%–96% with a short reaction time of ≤20 min while retaining its catalytic efficiency for five to six consecutive cycles. The exciting feature of this method is the easy access to pure products without chromatographic separation.

### Ionic Liquid-Based Catalysts

Ionic liquids are a brilliant alternative for the vintage volatile solvents, made of ionic components such as anions or cations with covalent crystalline structures. The only difference between molten salts and ionic liquids is the melting point which is presumed to be <100°C for ionic liquids, making them stable and efficient solvents. The low-volatility, low-melting-point, and low-vapour pressure impart heterogeneous characteristics to these ionic liquids aiding their hassle-free separation. The ionic components of the liquids account for their intense polarity, which validates their physical and chemical properties. Because of high solubility and eminent anti-corrosion activity, ionic liquids are used as anti-corrosion agents for metals and alloys and as a significant solvent catalyst in various industrial and laboratory preparations. Followed by the ammonium-hinged ionic liquid invention, researchers designed different ionic liquids incorporating imidazolium, pyridinium, phosphonium, and tetra-ammonium components.

In contrast to their ionic nature, ionic liquids are poor conductors of electricity and can dissolve polar and non-polar materials. Ionic liquids are differentiated depending on the functional groups of the side chains, which act as electron-exchangeable sites, where the electron-donating groups impart basic nature. In contrast, electron-withdrawing nature imparts an acidic nature to the liquids.

**SCHEME 25:**
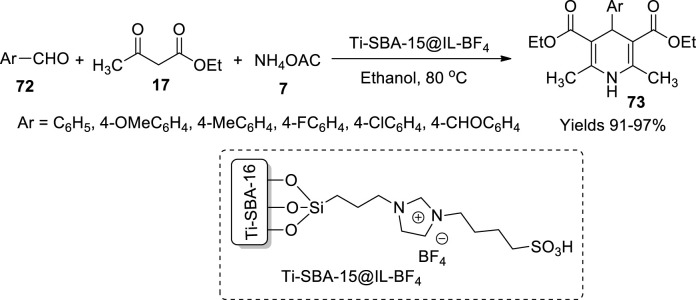
Synthesis of dihydropyridines by the Ti-SBA-15@IL-BF_4_ catalyst.

Tan and his colleagues (Tan et al., 2019) have formulated a technique for incorporating titanium into a mesoporous material to catalyse Hantzsch reaction mediated by an ionic liquid (Scheme 25). The tailored catalyst Ti-SBA-15@IL-BF_4_ was used to form 1,4-dihydropyridine derivatives (**73**) which showed high yielding capacity and sustained its catalytic ability even after running for five consequent cycles. The catalytic activity is aided by the mesoporosity of the material and the acidic nature of the ionic liquid with a product yield that gained excellent yields around 93% within a short reaction time.

**SCHEME 26:**
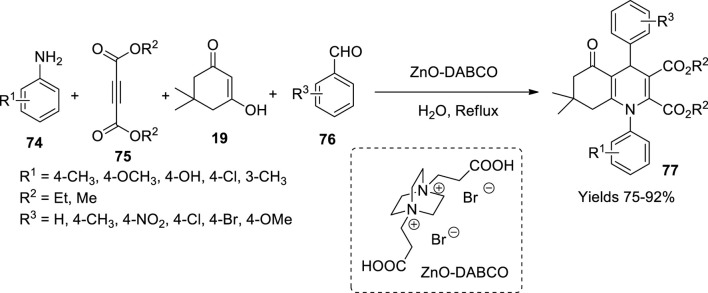
Synthesis of hydroquinolines by the ZnO-DABCO catalyst.

Mondal and her colleagues (Mondal et al., 2019) fabricated quasi-heterogeneous DABCO-based ZnO nanoparticles mediated by the acidic ionic liquid using the solvothermal technique. DABCO is a 3-amine with a localised electron lone pair of nitrogen in a cage-like structure. The DABCO is di-substituted with the carboxylic group when reacted with 3-bromopropionic acid. The combined size of the catalyst particle was 200–400 nm, whereas the size of the ZnO NP was 5–6 nm offering a greater surface area-to-volume ratio. The magnificence of this catalyst lies in the fact that it carries acidic moiety imparted by the ionic liquid apart from the basic nature inherited by the amorphous zinc oxide; hence, it possesses both acidic and basic nature in a single structure. They analysed the catalytic performance of synthesising hydroxyquinoline derivatives (**77**), namely, N-aryl poly-hydro quinoline-based drugs, which resulted in excellent yields as high as 91% within a minimum reaction time of 2 h using water as a solvent (Scheme 26).

**SCHEME 27:**
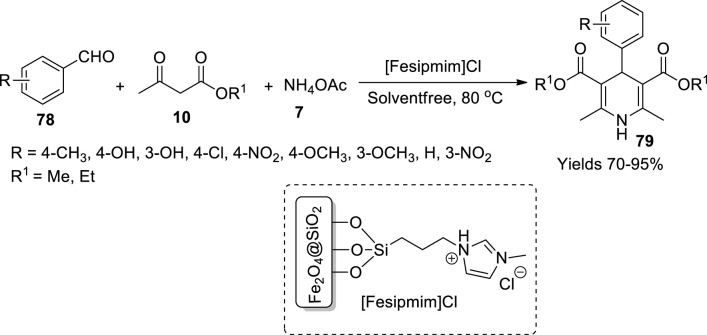
Synthesis of dihydropyridines by the [Fesipmim]Cl catalyst.

Agarwal and Kasana synthesised ferromagnetic nanocomposite, [Fesipmim]Cl, by the sol–gel method, and used it as a catalyst for generating dihydropyridines (**79**) *via* the Hantzsch reaction (Agrwal and Kasana, 2020). To prepare the desired nanocomposite, initially, a 1:2 ratio of Fe^+2^ and Fe^+3^ was dispersed in 200 ml of water, while vigorously stirring 25 ml of ammonium solution (30%) which was added and further subjected to mechanical stirring for 24 h to obtain black nanoparticles, which were washed and dried at 80°C. The gathered nanoparticles were laminated with silica employing the sol–gel method. A volume of 1.2 ml of tetraethyl orthosilicate is introduced into a nanoparticle-dispersed solution of ethanol, water, and ammonia while constantly stirring for 12 h. The obtained ferrite@SiO_2_ nanoparticles were washed and dried. The dried nanoparticles were gathered and dispersed in water. Simultaneously, ionic liquid, [Sipmim]Cl, was also prepared by refluxing 4.8 ml of 0.06 mol 1-methylimidazole and 14.5 ml of 0.07 mol (3-chloropropyl) triethoxysilane. Later, 2 g of ferrite@SiO_2_ NPs and 25 ml of dry toluene were mixed in a beaker, and 1 g of [Sipmim]Cl was introduced dropwise into it. The obtained nanoparticles were washed and dried at 60°C for 2 h. The size catalyst nanoparticle was 7–11 nm from TEM analysis, and the catalyst’s magnetisation was assessed to be 13 emu/g from VSM data. Its catalytic efficiency was investigated on the three-component Hantzsch reaction involving benzaldehyde (**78**), ethyl acetoacetate (**10**), and ammonium acetate (**7**) (Scheme 27). A good 95% yield of dihydropyridines within a solvent-free abode with a minimum catalyst (100 mg) and a short reaction time was observed. The ferrite@SiO_2_ NPs sustained their vitality for seven consequent cycles.

**SCHEME 28:**
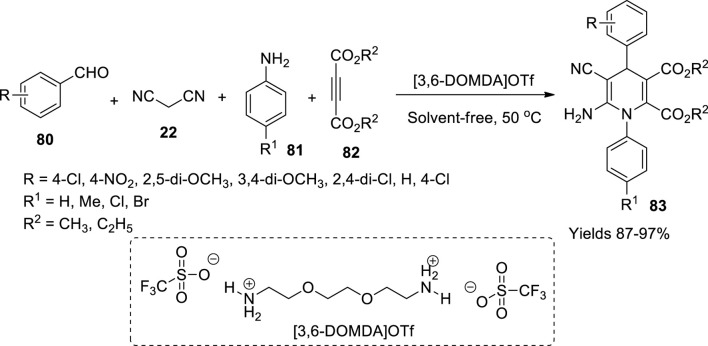
Synthesis of dihydropyridines by the [3,6-DOMDA]OTf catalyst.

Baghery and Zolfigol have reported the ability of 3,6-dioxaoctamethylenediamminium trifluoromethanesulfonate, a convenient ionic liquid, as a catalyst for synthesising 1,4-dihydropyridines (**83**) (BagheryZolfigol and Zolfigol, 2017). To prepare the ionic liquid, 6 mmol of trifluoromethanesulfonic acid was dropwise added to 3 mmol of 3,6-dioxaoctamethylenediamine at 5°C under stirring. Stirring continued for 25 min at room temperature. The acquired yellow-coloured product was washed with diethyl ether to decant the ruminants and vacuum dried at 50°C for 1 h. FT-IR, ^1^H, ^13^C and ^19^F NMR, MS, TG, difference thermo gravimetric (DTG), and difference thermo analysis (DTA) data were used to characterise the material. The single-pot four-component reaction involved substituted aldehyde (**80**), malononitrile (**22**), acetylene dicarboxylates (**82**), and anilines (**81**) in equal 1-mmol quantities in solvent-free abode at 50°C. 3,6-Dioxaoctamethylenediamminium trifluoromethanesulfonate showed remarkable activity. The reaction was assumed to follow Knoevenagel condensation along with Michael addition and intramolecular cyclisation. The yield of 1,4-dihydropyridines was high, around 93%–96%, with minimal use of a catalyst (2–5%) within 30 min (Scheme 28). The product was confirmed by using FT-IR, ^1^H, and ^13^C NMR. The catalyst sustained its activity even after six consecutive cycles.

**SCHEME 29:**
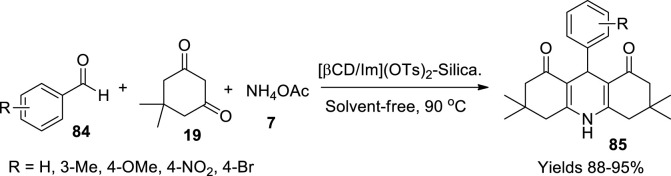
Synthesis of hydroquinolines by silica supported ionic liquid catalyst.

Moheiseni *et al.* (Moheiseni et al., 2021) designed a dicationic liquid bolstered with β-cyclodextrin/imidazolium, which was adhered to a silicon gel. The composite was used as a facile catalyst in the condensation of the Hantzsch reaction. The preparation of the catalyst involved several steps. Initially, mono-Ts-βCD was prepared using a solution of β-cyclodextrin taken in NaOH dissolved in 10 ml of distilled water. The temperature of the mixture was dropped to 0°C–5°C while adding THF and continuously stirred. While maintaining the same temperature range, a solution of TsCl in THF was added dropwise while stirring. The reaction mixture was neutralised using 2 N HCl and later poured into ice to obtain a white-coloured precipitate filtered and washed with acetone. Simultaneously, a reaction mixture of imidazole and NaOH was added to 10 ml of DMSO and stirred at 60°C while adding 1,4-dibromobutane. The mixture was allowed to drop its temperature and poured into water to obtain 1,4-bis(imidazol-1-yl)-butane as a white solid. [βCD/Im](OTs)_2_ was prepared by mixing solutions of mono-Ts-βCD and 2.5 ml of dimethylformamide (DMF) into a mixture of 1,4-bis(imidazol-1-yl)-butane and 2.5 ml of DMF while maintaining the temperature between 40°C and 60°C. The white precipitate obtained was filtered and washed with CCl_4_. To adhere the prepared ionic product onto silica gel, to the solution containing [βCD/Im](OTs)_2_ in 30 ml of DMF and NaOH, 3-chloro propyl trimethoxysilane was added slowly under continuous stirring and temperature maintained at 90°C in an N_2_ environment after 30 min. The product was filtered and added to a solution containing tetraethoxysilane and 1-butanol in 0.7 ml of water. The mixture was neutralised using 2 M HCl. The hydrogel was stirred continuously for 3 h at 80°C to obtain a final white precipitate filtered and washed with lukewarm water and acetone. The gathered catalyst was characterised by FTIR, SEM, and NMR using TMS as a standard. The authors established the catalytic efficiency of the di-ionic liquid on a single-pot Hantzsch condensation reaction between aromatic aldehyde (**84**), a β-dicarbonyl compound (**19**), and ammonium acetate (**7**) at a temperature of 90°C to obtain the product, hydroquinolines (**85**). The product was filtered and recrystallised in ethyl alcohol (Scheme 29). The catalyst showed high efficiency with 0.02 g of the catalyst offering a high 90% yield at 90°C in 20 min, which is relatively high compared with Tripodo’s technique which gives 35%.

### Miscellaneous Catalysts

**SCHEME 30:**
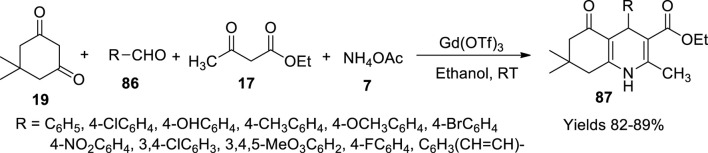
Synthesis of hydroquinolines by the Gd(OTf)_3_ catalyst.

Mansoor *et al.* (Sheik Mansoor et al., 2017) observed the advantageous characteristics of gadolinium(III) trifluoromethanesulphonate {Gd(OTf)_3_} to synthesise polyhydroquinolines (**87**) at room temperature. It showed significant potential to perform ideally in both polar and nonpolar solvents, making it a desirable catalyst. Gd(OTf)_3_ acted efficiently as a mild Lewis acid catalyst for the four-component coupling reaction between benzaldehyde (**86**), an active methyl group (ethyl acetoacetate) (**17**), 5,5-dimethyl-1,3-cyclohexanedione (dimedone) (**19**), and ammonium acetate (**7**) (Scheme 30). After confirming the completion of the reaction through TLC, the obtained product was recrystallised for purification. A good yield (82%–89%) of the target product was achieved at a minimal catalytic use as small as 1 mol%. Good retention of the catalytic activity was observed at 5 mol% using ethanol as solvent. Other solvents like acetone generated undesirable by-products due to rapid reactivity accompanied by the usage of a catalyst. The catalyst was retrieved comfortably from the filtrate, aiding its slightly higher potential to be soluble in water than organic solvents. The filtrate was subjected to evaporation under reduced pressure to gather the catalyst for further usage.

**SCHEME 31:**
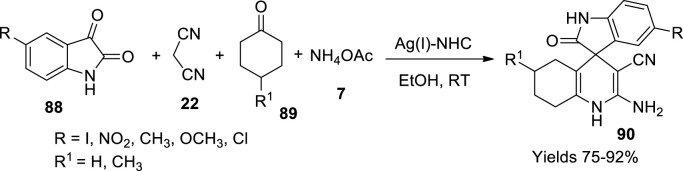
Synthesis of spiro oxindole-hydroquinolines by the Ag-NHC catalyst.

Balaboina and his colleagues (Balaboina et al., 2019) have prepared spiro oxindole-hydroquinolines (**90**), which paves a contemporary pathway for the drugs against cancer cells employing a single-pot method aside from Ag(I) and organo-*N*-heterocyclic carbenes used as a catalyst. This new catalyst was a progeny from labile Ag(I)-NHC in ethanol; therefore, it accommodates acidic Ag(I) alongside a basic NHC. A 3%–5% metal to organic–complex ratio was maintained for a smooth reaction. A single-pot four-component reaction between isatin (**88**), malononitrile (**22**), cyclo ketones (**89**), and ammonium acetate (**7**) was utilised (Scheme 31). The formation of spiro oxindole was established by TLC, EA, ESI-Mass, ^1^H, and ^13^C-NMR spectroscopic techniques apart from the MP value and XRD data. It was assumed to follow Knoevenagel condensation along with Michael addition yielding a high amount of product around 91% within 10 min. The prepared drugs were studied for their combating capacity toward two types of cancer cells, namely, MCF-7 and HepG2, which showed appreciable cytotoxicity.

**SCHEME 32:**
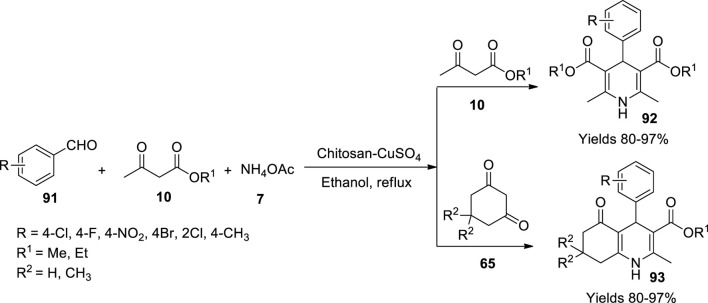
Synthesis of dihydropyridine and hydroquinolines by the chitosan-CuSO_4_ catalyst.

Dekamin and his team (Dekamin et al., 2016) have explored a chitosan macromolecular-based CuSO_4_ catalyst to synthesise Hantzsch products. A volume of 5 g of chitosan in 100 ml of water was dispersed, to which 1 g of CuSO_4_ was added under continuous stirring. The desired catalyst was isolated upon centrifuging the resultant mixture after 2 h. The catalyst was characterised using FT-IR, ICP-AES, FESEM, and EDX. 1,4-Dihydropyridines (**92**) and phenyl hydroxyquinoline derivatives (**93**) were synthesized by refluxing a mixture of aldehydes (**91**), β-dicarbonyls (**10**), and ammonium acetate (**7**) in an ethanolic medium; the catalyst facilitated high 97% yields with minimal loading of 0.02 g within a reaction time of 15 min (Scheme 32). The reaction pathway was assumed to follow Knoevenagel condensation along with Michael addition and tautomerism. The hygroscopic nature of chitosan assisted in the adsorption of water molecules allowing better interaction which promotes activation of carbonyl compounds.

**SCHEME 33:**
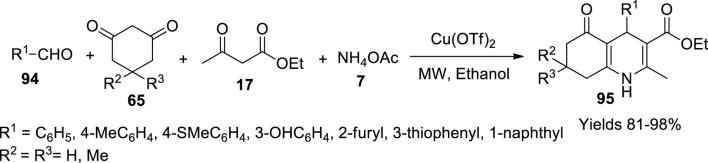
Synthesis of hydroquinolines by the Cu(OTf)_2_ catalyst.

Pasunooti and his colleagues (Pasunooti et al., 2010) have explored a single-pot multicomponent synthetic method for hydroquinolines (**95**) using a copper catalyst under microwave irradiation. Cupric (II) trifluoromethanesulphonate [Cu(OTf)_2_] was perceived to be a promising catalyst to obtain a high yield around 90% with a minimal amount of catalytic molar percentage, i.e., 2% at 100°C within 15 min of reaction time (Scheme 33). They optimised the Hantzsch reaction conditions, using benzaldehyde (**94**), ethyl acetoacetate (**17**), dimedone (**65**), and ammonium acetate (**7**) along with ethyl alcohol and Cu(OTf)_2_. The components were irradiated under microwave to achieve 1,4-dihydropyridines.

**SCHEME 34:**
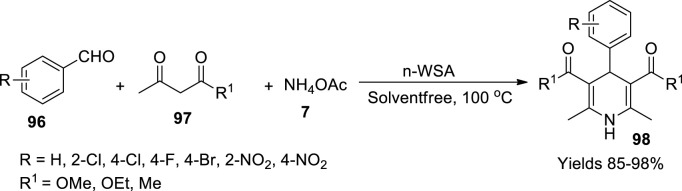
Synthesis of dihydropyridines by the nano-WO_3_ catalyst.

Bitaraf *et al.* (Bitaraf et al., 2016) appraised the significance of tungsten trioxide-bolstered sulphonic acid nano (n-WSA) composites in the preparation of 1,4-dihydropyridines (**98**). This nanocatalyst was prepared using powdered nano-WO_3_, and dichloromethane took in a suction flash connected to a pressure dropping funnel and a gas inlet tube facilitating the conduction of HCl gas over the water adsorbent. Chlorosulphonic acid was introduced into it dropwise, which resulted in the evolution of HCl gas. After the HCl gas ceased, the reaction mixture was stirred continuously for 30 min. The unreacted dichloromethane was removed under reduced pressure to obtain a dark green powder, washed with ethanol, and dried for 6 h at 120°C. The FE-SEM images suggest that the particle size of the nanocatalyst is around 60–70 nm. A single-pot three-component reaction involving aromatic aldehydes (**96**), β-dicarbonyls (**97**), and ammonium acetate (**7**) in a solvent-free abode in the presence of n-WSA was considered (Scheme 34). The method was presumed to follow Knoevenagel condensation along with Michael addition. The catalyst showed promising performance with an excellent yielding capacity of 98% within 15 min of reaction time. The reaction parameters were optimised under a central metal composite design.

**SCHEME 35:**
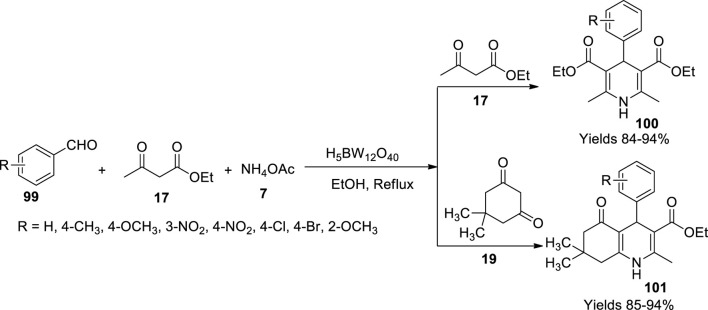
Synthesis of dihydropyridine and hydroquinolines by the H_5_BW_12_O_40_ catalyst.

Momeni and his colleagues (Momeni et al., 2020) developed a routine to synthesise 1,4-dihydropyridines (**100**) and polyhydro quinolines (**101**), employing H_5_BW_12_O_40_ as a novel catalyst. They implemented a single-pot Hantzsch reaction mechanism using substituted aldehydes (**99**), acetoacetic esters (**17**), and ammonium acetate (**7**) refluxed using borotungstic acid (H_5_BW_12_O_40_) as a catalyst. The negative charge of the catalyst aids in its activity as a Bronsted acid. The resultants were assessed using TLC in combination with n-hexane and ethyl acetate in the ratio 7:3. The reminiscent was washed using ethyl acetate, and the products were dried using Na_2_SO_4_. The electron-withdrawing group, such as NO_2_ at aldehyde, was observed to gain the highest yield of 98% within 45 min (Scheme 35). The reaction was presumed to follow Knoevenagel condensation along with Michael addition. The gathered products were characterised using FT-IR, ^1^H, and ^13^C NMR spectral data.

**SCHEME 36:**
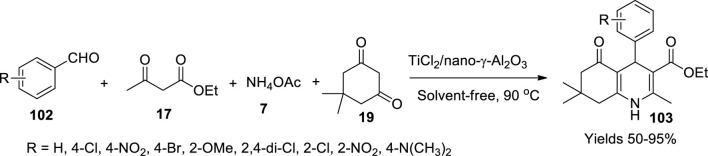
Synthesis of hydroquinolines by the TiCl_2_-Al_2_O_3_ catalyst.

Mirjalilil *et al.* (Mirjalili et al., 2018) used a Lewis acid catalyst, TiCl_2_/nano-γ-Al_2_O_3_, to prepare hydroxyquinoline derivatives. A volume of 60 ml of 1 M NaOH solution was initially added into a beaker containing 66 g of Al_2_(SO_4_)_3_.18H_2_O and stirred vigorously to obtain a white precipitate of Al(OH)_3_, which was washed with distilled water and dried. A volume of 20 g of Al(OH)_3_ was dissolved in 100 ml of 1 M NaOH, and 3% (v/v%) polyethylene glycol was added. The pH of the mixture was adjusted to eight and subjected to vigorous stirring to obtain a solid separated by centrifugation. The solid was collected and washed using distilled water and calcinated at 800°C for 3 h to get nano-γ-Al_2_O_3_. To 1 g of nano-γ-Al_2_O_3_ dissolved in 10 ml of dichloromethane, 0.5 ml of TiCl_2_ was added dropwise. The resulting solution was vigorously stirred till precipitation. The residue separated by filtration was washed using CHCl_3_ and dried at room temperature. The SEM revealed its amorphous shape with 50 nm of particle size and a surface area of 75.6 m^2^/g through BET analysis. The material was found stable up to 400°C. The catalyst’s capacity was assessed in the preparation of hydroquinolines (**103**) in a single-pot reaction method using 1 mmol of aryl aldehyde (**102**), 2 mmol of 1,3-dicarbonyl ester (**17**), and 1.5 mmol of ammonium acetate (**7**) in the presence of 0.05 ml of TiCl_2_/Nano-γ-Al_2_O_3_ in a solvent-free abode (Scheme 36). The product obtained was filtered and appraised using FT-IR, ^1^H, and ^13^C NMR spectroscopic methods. Aldehyde substituted with the +I group gained a higher yield of around 95% within 26 min.

**SCHEME 37:**
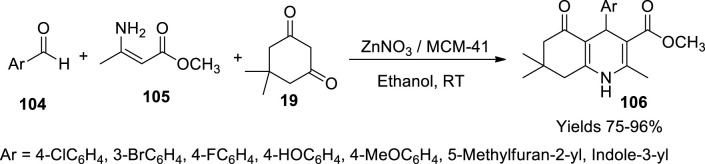
Synthesis of hydroquinolines by the Zn-MCM-41 catalyst.

Oskuie and his colleagues (Oskuie et al., 2020) worked on the catalytic preparation of the Hantzsch reaction to produce polyhydroquinolines (**106**) catalysed by Zn/MCM-41. The impregnation method was used to induce ZnNO_3_ onto MCM-41. The vintage Hantzsch reaction mixture consisting of aryl aldehyde (**104**), dimedone (**19**), and methyl-3-aminocrotonate (**105**) was employed in the presence of the prepared catalyst in a solvent-free environment. The reaction proceeded by activating the carbonyl groups through Zn^+2^ for Knoevenagel condensation, and the enamine intermediate participates in Michael addition. The acquired product was filtered and dried. High yields were achieved by using 3- and 4-fluorobenzaldehyde, salicylaldehyde, and 4-hydroxybenzaldehyde in combination with methyl 3-aminocrotonate and 5-methyl furfural, while the lowest yield was attained with 3-indole-carboxaldehyde (Scheme 37). The highest 97% yield was observed within a reaction time of 2 h. The obtained product was explored using FT-IR, ^1^H, and ^13^C NMR spectroscopic analysis. Cytotoxicity of the prepared polyhydroquinolines was assessed against MCF 7, SK BR-3, and HT 29 cancer cell lines employing cell cytotoxicity assay by MTT to determine their IC_50_ values 2.5 mg/cm^3^ and 180 mm^3^ RPMI value.

**SCHEME 38:**
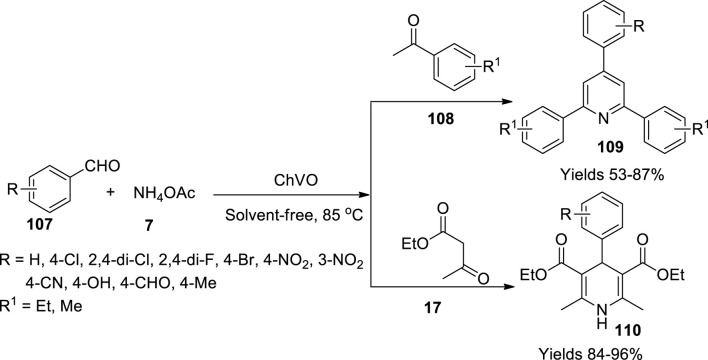
Synthesis of dihydropyridine and triaryl pyridines by the chitosan vanadium catalyst.

Safaiee and his coworkers (Safaiee et al., 2018) have constructed a vanadium oxo catalyst bolstered on chitosan and effectively used to synthesise series of 1,4-dihydropyridines (**110**) and 2,4,6-triarylpyridines (**109**) adopting anomeric-based oxidation. Chitosan was refluxed with vanadium pentoxide in water to form a black precipitate after 24 h, which was filtered and washed with hot DM water followed by drying it at 80°C. By FT-IR, XRD, SEM, EDX, ICP-MS, and TGA analyses, the material was characterised. XRD amorphous structure SEM and wavelength-dispersive X-ray analysis (WDX) confirmed the presence of V, N, O, and C atoms. Tg and DTG stability of the catalyst was at 225°C. The reaction mixture of aldehyde (**107**), *β*-ketoester (**17**), and ammonium acetate (**7**) in the presence of 5 mg of ChVO catalyst in a solvent-free abode at 80°C gave 1,4-dihydropyridines in good yields (Scheme 38). The obtained product was filtered and dissolved in hot ethyl acetate to isolate the catalyst and refiltered to retrieve the heterogeneous catalyst. The product solution was evaporated, washed, and recrystallised from a solution mixture of ethanol and water taken in the ratio of 1:10. In the other reaction, a mixture containing aromatic aldehydes, acetophenone derivatives, and ammonium acetate were reacted in the presence of 5 g of the ChVO catalyst to obtain 2,4,6-triarylpyridines at a temperature of 130°C in a hot oil bath. Hot ethanol was added to the reaction solution to dissolve the product formed and isolate the heterogeneous catalyst. The reaction solution was evaporated and recrystallised to gather 2,4,6-triarylpyridines. As per TG and DTA analyses, the catalyst material is stable up to 225°C. The amorphous structure of the catalyst was represented by SEM images, whereas the WDX images gave a note about the elements present in the catalyst. The TLC method concluded the reactions with a medium containing n-hexane and ethyl acetate in the ratios 3:7 and 4:15. The obtained derivatives of 1,4-dihydropyridines and 2,4,6-triarylpyridines were examined using FT-IR, ^1^H, and ^13^C NMR analyses. The use of the ChVO catalyst greatly enhanced the yield (89%) while reducing the reaction time to 45 min. The retrieved catalyst retained its viability and catalytic potential for several consequent cycles.

**SCHEME 39:**
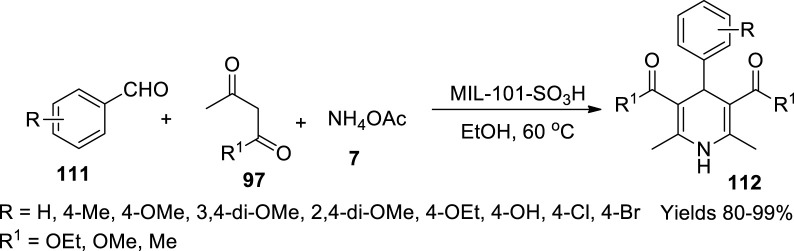
Synthesis of dihydropyridines by the MIL-101-SO_3_H catalyst.

Devarajan and Suresh (Devarajan and Suresh, 2019) studied the performance of a solid heterogeneous Bronsted acid catalyst, MIL-101-SO_3_H metal-organic framework, in the synthesis of 1,4-dihydropyridines (**56**) using Hantzsch reaction. Initially, a reaction mixture of monosodium 2-sulfoterephthalic acid and CrO_3_ was dissolved in a solution of 0.53 ml of HCl and 20 ml of water and autoclaved at 180°C for 168 h. It was allowed to drop to room temperature and filtered. The product was washed with 400 ml of DM water and 100 ml of methanol. The green powder was dissolved in DMF, heated at 120°C for 24 h, and again added to a solution of aqueous methanol at the same temperature to achieve microcrystalline powder. The collected green powder was dissolved in a solution containing 0.08 M of HCl. The obtained product was treated with aqueous methanol for three consecutive cycles. Finally, the green powder was washed with water and vacuum dried at 120°C. It showed a crystalline structure with 1,497 m^2^/g of surface area, as XRD represented and BET values. The MIL-101-SO_3_H aggregation was explored by SEM analysis. EDAX showed that the catalyst contains 13.47 wt% of chromium and 3.47% of sulphur which has stability up to 500°C. The catalytic potential was assessed by administering 20% (wt%) of MIL-101-SO_3_H MOF in Hantzsch single pot reaction, including aldehyde (**111**), 1,3-dicarbonyl compound (**97**), and ammonium acetate (**7**) to produce 1,4-dihydropyridines (Scheme 39). After the reaction completion, the catalyst was isolated by centrifugation and washed with ethanol repeatedly. The retrieved catalyst was heated with DMF at 100°C for 1 h. After removal of DMF by filtration, the material was dried at 120°C for 3 h. The catalyst gained a high 99% yield on sonication, and in addition to the excellent yield, the catalyst retained its viability and potential for several consecutive cycles.

**SCHEME 40:**
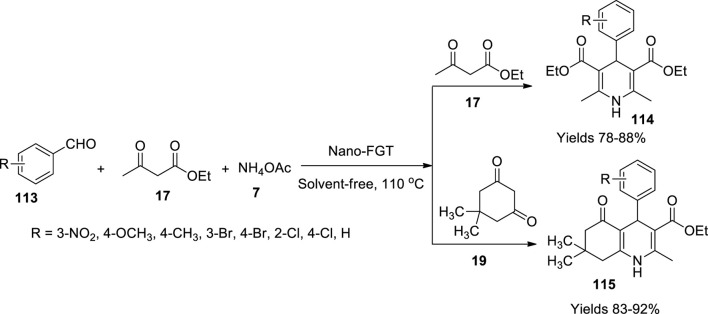
Synthesis of dihydropyridines by the glutathione catalyst.

Maleki *et al.* (Maleki et al., 2019c) have produced 1,4-dihydropyridines (**114**) and hydroquinolines (**115**) using a single-pot system under the influence of magnetic nanoparticles infused with glutathione. The desired catalyst was prepared by adding ferric oxide to water and methyl alcohol solution and subjecting it to sonication. A volume of 0.4 g of glutathione was added, and the mixture was further sonicated for 2 h. The magnetic material was separated using an external magnetic field, and the resulting solution was filtered by washing it with water and methyl alcohol and oven-dried at 50°C–60°C. The adhesion of glutathione onto the magnetic particles was confirmed by various analytical methods such as FT-IR, XRD, TGA, TEM, VSM, EDX, and elemental analysis. The EXD images show the presence of Fe, O, N, and S. The catalytic activity and viability of the material were established using the Hantzsch reaction to synthesise 1,4-dihydropyridines (Scheme 40). The reaction mixture in a solvent-free environment consisted of benzaldehyde (**113**), ethyl acetoacetate (**17**), ammonium acetate (**7**), and dimedone (**19**). A Knoevenagel condensation followed by Michael addition generates the dihydropyridine derivative. The method offers a 90% yield within 35 min with minimal use of 0.02 g of the prepared catalyst.

**SCHEME 41:**
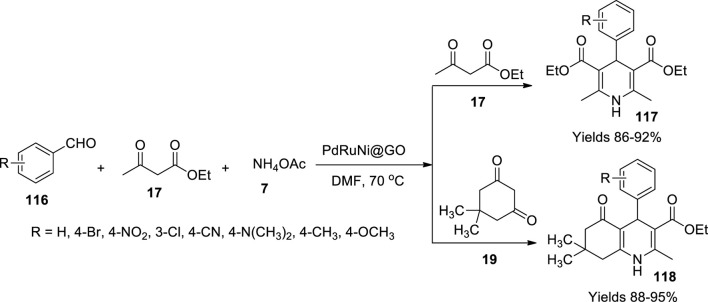
Synthesis of dihydropyridine and hydroquinolines by the PdRuNi@GO catalyst.

Demirci and the team (Demirci et al., 2016) investigated mono-dispersed PdRuni@GO prepared by the double solvent reduction method as a catalyst in Hantzsch reaction to prepare dihydropyridine (**117**) and hydroxyquinoline (**118**) derivatives in a single-pot system. A lysed mixture of 0.25 mmol of Pd, Ni, and Ru in dehydrated tetrahydrofuran in 0.25 mmol of octane thiol was subjected to ultrasonication till the solution turned black-brown, suggesting the formation of Pd–Ni–Ru nanoparticles which was vacuum dried. The gathered nanoparticles were combined with graphene oxide in an equal molar ratio and subjected to sonication to gain Pd-Ni-Ru@GO nanoparticles. The XRD values of the gathered nanoparticles describe their perovskite crystalline structure with 3.64 nm of diameter with a surface area of 136.2 cm^2^/g as represented by BET images. The catalytic performance of the prepared nanoparticles was assessed in preparing 1,4-dihydropyridines. The reaction mixture involved an equimolar amount of ammonium acetate (**7**), ethyl acetoacetate (**17**), and 1 mmol of aldehyde (**116**) suspended in 2 ml of DMF, plus 6 mg of Pd-Ni-Ru@GO nanoparticles as a catalyst, under 70°C for 45 min (Scheme 41). After the completion of the reaction, the mixture was centrifuged and poured into 20 ml of ice-cold water to form a vacuum filtered, washed, and dried precipitate. With substituted aldehydes, high yields of 93%–98% were obtained within a reaction time of 45 min. The gathered product was subjected to various spectroscopic analyses to elucidate the derivatives. The catalyst was gathered and reused for five consecutive cycles without losing its vitality when subjected to trial under ICP-OES analyses.

## Conclusion

The review highlighted the eminence of mainly the heterogeneous catalyst materials in preparing different novel dihydropyridine derivatives. The monograph described preparation techniques for various catalyst materials in detail. It covered facile and benign magnetic, silicon- and zirconium-bolstered, and ionic liquid-based heterogeneous catalysts, which can coherently facilitate excellent yields in short reaction times in a cost-effective and eco-friendly way. The target products are valuable molecules proficient in curing long-term disorders and drug delivery to the target tissue. The catalyst materials and protocols considered in this review are easy to handle, nontoxic, and easily prepared. High-temperature and pressure resistance ranges make them ideal and sustainable catalysts for preparing heterocyclic compounds, dihydropyridine in particular. These innovative pathways tailored for the synthesis of dihydropyridine derivatives have paved fresh approaches for further research.
